# Identification of Novel GANT61 Analogs with Activity in Hedgehog Functional Assays and GLI1-Dependent Cancer Cells

**DOI:** 10.3390/molecules29133095

**Published:** 2024-06-28

**Authors:** Dina Abu Rabe, Lhoucine Chdid, David R. Lamson, Christopher P. Laudeman, Michael Tarpley, Naglaa Elsayed, Ginger R. Smith, Weifan Zheng, Maria S. Dixon, Kevin P. Williams

**Affiliations:** 1 INBS PhD Program, North Carolina Central University, Durham, NC 27707, USA; daburabe@eagles.nccu.edu; 2 Biomanufacturing Research Institute and Technology Enterprise, North Carolina Central University, Durham, NC 27707, USA; lchdid@omniab.com (L.C.); davidlamson@yahoo.com (D.R.L.); cpl@laudeman.com (C.P.L.); mtarpley@nccu.edu (M.T.); nelsayed@eagles.nccu.edu (N.E.); grsmith@nccu.edu (G.R.S.); wzheng@nccu.edu (W.Z.); 3 Department of Pharmaceutical Sciences, North Carolina Central University, Durham, NC 27707, USA

**Keywords:** hedgehog, GLI1, GANT61, C3H10T1/2, high-throughput screening, GLI inhibitors, molecular docking

## Abstract

Aberrant activation of hedgehog (Hh) signaling has been implicated in various cancers. Current FDA-approved inhibitors target the seven-transmembrane receptor Smoothened, but resistance to these drugs has been observed. It has been proposed that a more promising strategy to target this pathway is at the GLI1 transcription factor level. GANT61 was the first small molecule identified to directly suppress GLI-mediated activity; however, its development as a potential anti-cancer agent has been hindered by its modest activity and aqueous chemical instability. Our study aimed to identify novel GLI1 inhibitors. JChem searches identified fifty-two compounds similar to GANT61 and its active metabolite, GANT61-D. We combined high-throughput cell-based assays and molecular docking to evaluate these analogs. Five of the fifty-two GANT61 analogs inhibited activity in Hh-responsive C3H10T1/2 and Gli-reporter NIH3T3 cellular assays without cytotoxicity. Two of the GANT61 analogs, BAS 07019774 and Z27610715, reduced *Gli1* mRNA expression in C3H10T1/2 cells. Treatment with BAS 07019774 significantly reduced cell viability in Hh-dependent glioblastoma and lung cancer cell lines. Molecular docking indicated that BAS 07019774 is predicted to bind to the ZF4 region of GLI1, potentially interfering with its ability to bind DNA. Our findings show promise in developing more effective and potent GLI inhibitors.

## 1. Introduction

GLI1 (glioma-associated oncogene homolog 1) was first identified in human glioma cancer cell lines as an oncogene [1] and subsequently shown to be a zinc finger (ZF) containing transcription factor [2,3]. GLI1 was shown to be a transcription factor that acts as the main effector of hedgehog (Hh) signaling [4,5], a major morphological pathway involved in development [6,7,8]. In mammals, GLI1 and its family members GLI2 and GLI3 all contribute to the transcription response to Hh activation (as recently reviewed in [6]). During canonical Hh signaling, the binding of the Hh ligand to its receptor, Patched 1 (PTCH1), relieves PTCH1 repression of Smoothened (SMO), a seven-pass transmembrane protein. This allows for the activation of SMO and the translocation of GLI proteins into the nucleus, turning on the transcription of various Hh target genes, including GLI1 itself. GLI1 activation creates a positive feedback loop, amplifying the overall Hh response [8].

Hh/GLI activation has been observed in various human tumor types [8,9,10,11]. This activation has been reported to occur through both canonical and non-canonical mechanisms, with GLI1 as the key transcription factor [12,13,14,15,16,17,18]). GLI1 expression has been associated with a poor prognosis and an advanced stage of various cancers [16,19]. GLI1 activation can occur through multiple pathways independent of SMO, including by PI3K/AKT/mTOR, TGF*β*, the DYRK family, and oncogenic drivers such as c-Myc [15,16,20,21,22]. Noncanonical GLI1 activation has been reported in esophageal [23], glioblastoma [24], and lung [25] cancers. Therefore, identifying drugs that target the Hh/GLI pathway has been a major focus in cancer drug development. Small-molecule inhibitors of SMO have shown the most success in targeting the Hh/GLI pathway [26,27], resulting in the development of three FDA-approved SMO inhibitors [28,29,30]. Despite the initial success of SMO inhibitors, clinical studies have reported resistance to these drugs [27,31]. This has led to identifying other potential drug targets in the pathway, including downstream at the level of GLI [20,32,33,34,35,36,37,38].

GLI1/DNA binding has been proposed as a druggable target [39]. GANT61, discovered by small-molecule screening in a *Gli* reporter cell-based assay [40], was shown to directly block GLI-mediated transcriptional activity [40]. Subsequent molecular docking and biochemical studies showed that GANT61 binds directly to GLI1 within the zinc finger (ZF) region [41]. Additionally, GANT61 has demonstrated anti-cancer activity in various cell studies, including the suppression of cancer cell growth [42,43] and the promotion of apoptosis and G1/S phase cell cycle arrest [44]. However, GANT61 was shown to be unstable under physiological conditions and hydrolyzes into an aldehyde derivative and a diamine (GANT61-D), which is the bioactive form that inhibits GLI activity [45]. Several other GLI-directed inhibitors have been identified [20,35,37,46,47]), including GANT58 [40], HPI-1-4 [48], glabrescione B [39], arsenic trioxide [49], genistein [50], pirfenidone [51], pyrvinium [52], FN1-8 [53,54], ketoprofen [55,56], nanoquinacrine [57], zerumbone [58], 5′-O-Methyl-3-hydroxyflemingin [59], and cynanbungeigenin C and D [60]. With the exception of arsenic trioxide, which is not a GLI-specific inhibitor [49,61], these GLI antagonists have not been clinically evaluated [37,46].

In our study, we aimed to identify novel and potent GLI antagonists with potentially improved physicochemical properties. Utilizing JChem for chemical structure searches, we identified fifty-two analogs similar to both GANT61 and its active form, GANT61-D. The screening of these fifty-two analogs in high-throughput Hh functional cell-based assays identified six potential inhibitors with potencies in the low micromolar range. Our qRT-PCR data confirmed that two analogs (BAS 07019774 and Z27610715) significantly inhibited *Gli1* mRNA expression in Hh-responsive C3H10T1/2 cells. Notably, BAS 07019774 inhibited the growth of GLI1-dependent glioma and lung cancer cells. Further, a computational molecular docking simulation predicted that BAS 07019774 binds to the ZF4 domain within the GLI1-DNA binding region. The amino acid residues interacting with BAS 07019774 are conserved between GLI1 and GLI2, suggesting a broader targeting potential. The identification of BAS 07019774 as a promising GLI antagonist provides a novel starting point for future research aimed at developing improved GLI-targeted compounds.

## 2. Results

### 2.1. Generating the GANT61 Analog Compound Set

**Figure 1:**
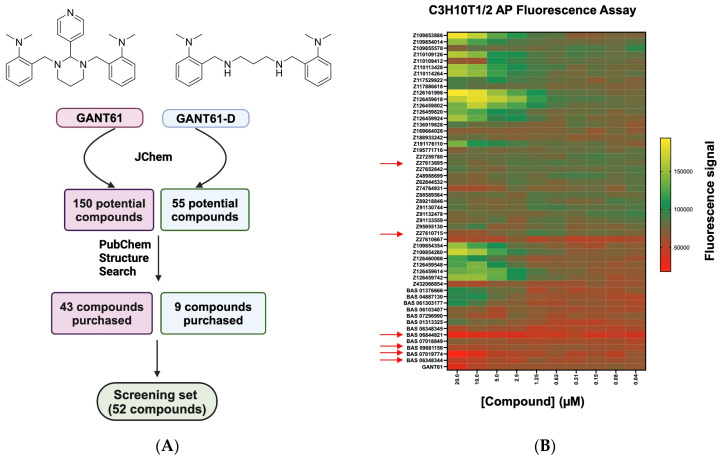
Screening of GANT61 analog compound set for hedgehog pathway inhibition. (**A**) Illustration of JChem and PubChem structure searches to identify compounds that share similar pharmacophores with GANT61 and the active form of GANT61 (GANT61-D). (**B**) Representative heat map profile for Hh pathway inhibitory effects of the GANT61 analog set in C3H10T1/2 cells. C3H10T1/2 cells were stimulated with SAG at its EC_50_ value (30 nM). GANT61 analogs were added in dose–response at the indicated concentrations, and after 5 days, alkaline phosphatase (AP) activity was measured using a fluorescent AP substrate. Each row represents dose–response data for a single GANT61 analog. The heat map key indicates that red stands for maximum inhibition and pale green stands for minimal inhibition. Red arrows indicate GANT61 analogs selected for further study.

To identify compounds structurally similar to GANT61 [40], we performed the Tanimoto 3D similarity search in Instant JChem (Chemaxon Ltd., Budapest, Hungary). This first search yielded 150 potential compounds (with coefficients ranging from 0.8 down to 0.5), and after assessing their availability, 43 compounds were identified for testing. Subsequent to our initial similarity search, it was reported that the active form of GANT61 was a diamine derivative (termed GANT61-D) resulting from the hydrolysis of the parent compound [45]. Hence, a second round of similarity searching using the GANT61-D chemical scaffold in JChem was performed. This search (with a coefficient >0.5) yielded 55 potential compounds, including GANT61. Many of these compounds overlapped with the initial search results with an additional nine novel candidates identified (Figure 1A). From the combined results of both searches, 52 GANT61 analogs were purchased from vendors as listed in Supplemental Table S1.

### 2.2. Assessing GANT61 Analogs for Inhibition of Hh Activity in the C3H10T1/2 Cell-Based Assay

**Figure 2:**
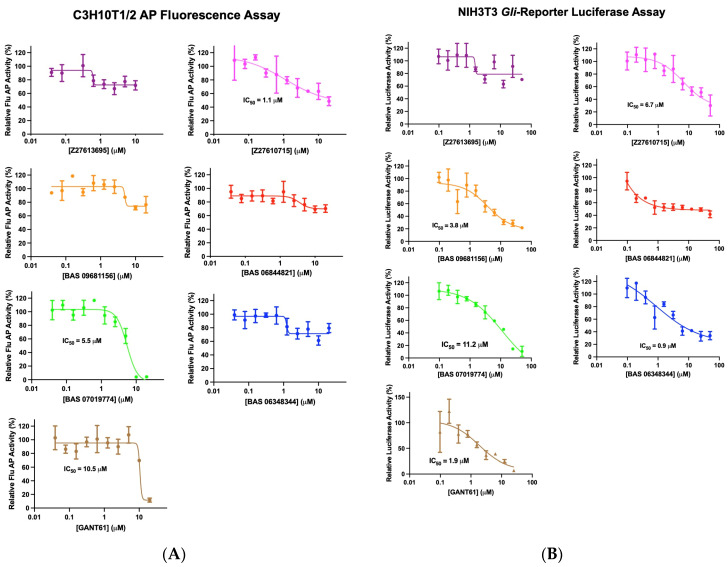
Dose–response curves for selected GANT61 analogs in Hh-responsive cellular assays C3H10T1/2 and *Gli* reporter NIH3T3. (**A**) The C3H10T1/2 AP fluorescence assay was carried out as in Figure 1. Data were normalized to SAG-stimulated cells and are displayed as the mean of % activity (*n* = 3 independent experiments). (**B**) *Gli* reporter NIH3T3 cells were treated with SAG at its EC_50_ value (26 nM), GANT61 analogs added in dose–response at the indicated concentrations, and cells were incubated at 37 °C for 30 h. Firefly luciferase activity was measured as described in Methods. Data were normalized to cells treated with SAG (negative control) and are displayed as the mean of % luciferase activity ± SD (*n* = 3 independent experiments). Dose–response curves were generated using non-linear regression, and IC_50_ values were determined using GraphPad 9.

The GANT61 analog set was first assessed for Hh pathway inhibition using the Hh-responsive C3H10T1/2 cell line assay, a well-accepted bioassay to evaluate inhibitors for this pathway [62,63]. In these murine pluripotent cells, Hh activation leads to alkaline phosphatase (AP) induction, a marker for differentiation into osteoblast lineages [64,65]. Compounds underwent high-throughput screening using an automated and optimized 384-well format with either a fluorescence or absorbance readout for AP activity. To induce Hh pathway activity, C3H10T1/2 cells were treated with SAG at its EC_50_ concentration (30 nM) (Supplemental Figure S1A) followed by GANT61 analog compounds added in dose–response. Cells were incubated for a further 5 days, and then the AP activity was measured. This initial screening of the fifty-two GANT61 analogs identified several compounds that decreased SAG-induced AP activity in the C3H10T1/2 cells (the fluorescence (flu) readout is shown in Figure 1B and the absorbance (abs) readout in Supplemental Figure S2A). Compounds with activity in only one readout (flu or abs) were excluded to eliminate those potentially interfering with the assay [66], resulting in six compounds being selected for further analysis. These six compounds were evaluated to determine their potency in C3H10T1/2 cells with the flu AP readout. Two analogs, BAS07019774 (IC_50_ = 5.5 μM) and Z27610715 (IC_50_ = 1.1 μM), exhibited stronger inhibitory activity compared to that of GANT61 (IC_50_ = 10.5 μM) (Figure 2A, Table 1). The remaining four analogs showed incomplete inhibition and did not reach 50% inhibition at the highest concentration tested. In the C3H10T1/2 colorimetric assay, the six GANT61 analogs showed inhibition profiles (Supplemental Figure S2B) comparable to those we observed in the fluorescence-based version of the assay (Figure 2A), with BAS07019774 and GANT61 again having full dose–response curves, yielding IC_50_ values of 3.3 μM and 3.7 μM, respectively (Table 1).

For further confirmation, the six GANT61 analog compounds were repurchased and retested for inhibition in the C3H10T1/2 assay using SAG and as an additional control, purmorphamine (PUR) [67,68], another Smo agonist to activate the Hh pathway (PUR, EC_50_ = 500 nM; Supplemental Figure S1A). The Hh-pathway-inhibitor KAAD-cyclopamine (KAAD-cyc) [69] was used as a positive inhibitor control (IC_50_ = 6.2 nM; Supplemental Figure S1A). The six GANT61 analogs displayed comparable inhibition profiles and IC_50_ values regardless of which Smo agonist (SAG or PUR) was used (Supplemental Figure S3). For Z27610715, BAS07019774, and GANT61, IC_50_ values of 8.1, 6.5, and 2.5 μM, respectively, were determined when using PUR as the pathway activator (Table 1).

### 2.3. Testing Selected GANT61 Analogs for Inhibition in the Gli-Luciferase Reporter NIH3T3 Cell Line

To confirm Hh pathway inhibition and to eliminate any cell-line-specific effects, we next tested the six identified GANT61 analogs in an orthogonal Hh-responsive cell line model, the Gli-luciferase reporter NIH3T3 cell line (BPS Bioscience, San Diego, CA, USA). In these engineered NIH3T3 cells, the gene for firefly luciferase was stably integrated to be under the transcriptional control of Gli-responsive elements. To assess the six GANT61 analogs, the Gli-luciferase NIH3T3 cells were stimulated with SAG at its EC_50_ concentration (26 nM, Supplemental Figure S1B), compounds added in dose–response (with KAAD-cyc as a positive control inhibitor (IC_50_ = 8.8 nM); Supplemental Figure S1B), and the luciferase activity measured. Four of the six GANT61 analogs significantly decreased the luciferase expression in a dose-dependent manner with Z27610715, BAS 09681156, BAS 07019774, and BAS 06348344 having IC_50_ values of 6.7, 3.8, 11.2, and 0.9 μM, respectively, and GANT61 having an IC_50_ value of 1.9 μM (Figure 2B, Table 1). BAS 06844821 had more modest effects on the luciferase activity, decreasing the activity by ~40% at the highest dose tested. As Z27613695 showed no significant inhibition of activity in any of the Hh assays (Table 1), this compound was no longer included for subsequent testing. The results from both of the Hh-responsive assays showed BAS 07019774 and Z27610715 were the most active (Table 1).

### 2.4. Assessing Selected GANT61 Analogs for Cell Cytotoxicity in the Hh-Cell-Based Assays

**Figure 3:**
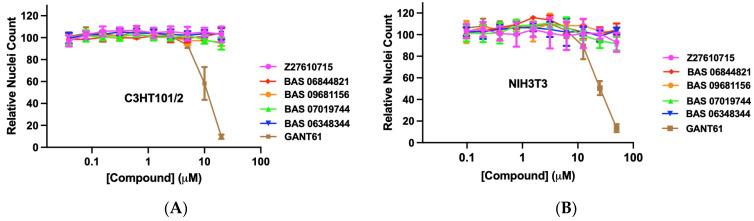
Effect of the selected GANT61 analogs on cell cytotoxicity in C3H10T1/2 and *Gli* reporter NIH3T3 cells. GANT61 analogs were added in dose–response at the indicated concentrations to C3H10T1/2 cells for 5 days (**A**) or to *Gli* reporter NIH3T3 cells for 30 h (**B**). Cells were then incubated with Hoechest-33342, and cell numbers were determined using high-content imaging as described in Methods. Data were normalized to SAG-treated cells. Data are displayed as the mean of the relative nuclei count ± SD (*n* = 3 independent experiments).

Next, we assessed whether the GANT61 analogs specifically inhibited Hh signaling or caused general cytotoxicity in the C3H10T1/2 and Gli reporter NIH3T3 cells. These cells were stimulated with SAG, selected GANT61 analogs added in dose–response, and the effects on cell number determined using the nuclear stain Hoechst 33342 [70]. The GANT61 analogs did not significantly affect the C3H10T1/2 cell number, while GANT61 significantly decreased the cell number at the highest concentration of 20 μM (a 93.5% reduction) (Figure 3A). Likewise, no significant reduction in Gli reporter NIH3T3 cell numbers was observed with the GANT61 analogs tested up to a concentration of 50 μM (Figure 3B). However, GANT61 showed significant cytotoxicity in these cells with cell count reductions of 49.4% and 86.5% at 25 μM and 50 μM, respectively (Figure 3B). These findings suggest that the inhibitory effects of the GANT61 analogs in both Hh-responsive cell lines are mediated through the Hh pathway rather than by a general cytotoxic effect.

### 2.5. Testing GANT61 Analogs for Inhibition of SAG-Induced Gli1 mRNA Expression in C3H10T1/2 Cells

**Figure 4:**
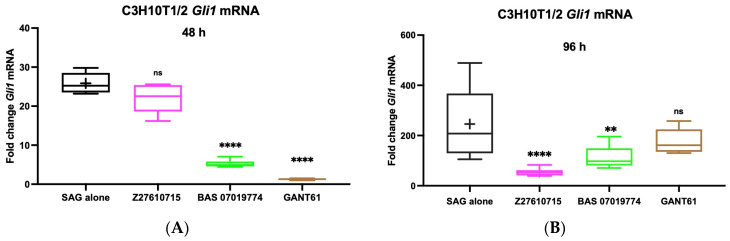
Effect of GANT61 analogs on *Gli1* mRNA expression in C3H10T1/2 cells. C3H10T1/2 cells were seeded in 12-well plates at a cell density of 60,000 cells/well, treated with SAG (30 nM) alone (control) or with SAG in the presence of 10 μM of BAS07019774, Z27610715, or GANT61. RNA was collected at 48 (**A**) and 96 (**B**) h, and *Gli1* mRNA expression was determined by Taqman qRT-PCR. Data were normalized to the housekeeping gene β-actin. The mean of three independent experiments ± SD is shown. Data were evaluated by one-way ANOVA followed by Tukey’s multiple comparisons test using GraphPad Prism 9. Significant differences relative to treatment of SAG alone (**** *p* < 0.0001, ** *p*-value = 0.0022, ns = not significant).

To determine the direct inhibition of Hh pathway transcriptional activity by the GANT61 analogs, Gli1 mRNA expression was measured in SAG-induced C3H10T1/2 cells. A preliminary time course assessed Gli1 mRNA expression in C3H10T1/2 cells induced by SAG, with two time points selected for further studies (48 and 96 h, Supplemental Figure S4A). Based on their consistent inhibitory activity in the previous Hh-responsive assays (Table 1), we selected the GANT61 analogs Z27610715 and BAS 0701977 for continued testing. C3H10T1/2 cells were stimulated with SAG at its EC_50_ concentration (30 nM) and treated with the two GANT61 analogs, and the Gli1 mRNA levels were measured using TaqMan qRT-PCR. As expected, SAG significantly increased Gli1 mRNA expression at 48 h (27-fold) and 96 h (278-fold) in comparison to that of untreated cells (Figure 4A,B). The positive control inhibitor, KAAD-cyc, effectively inhibited SAG-induced Gli1 mRNA expression (with a six-fold decrease at 48 h; Supplemental Figure S4B). BAS 07019774 significantly reduced Gli1 mRNA expression at both time points (4.9-fold at 48 h and 2.2-fold at 96 h) compared to the SAG-treated cells (Figure 4A,B), while Z27610715 showed a significant reduction only at the later time point (4.5-fold at 96 h) (Figure 4B). Interestingly, GANT61 reduced Gli1 mRNA expression at 48 h (20.4-fold) but did not significantly reduce Gli1 mRNA expression at 96 h (Figure 4B). These findings further support the inhibitory activity of the GANT61 analogs on the Hh pathway.

### 2.6. Effects of GANT61 Analogs on the Viability of Glioblastoma and Lung Cancer Cell Lines

**Figure 5:**
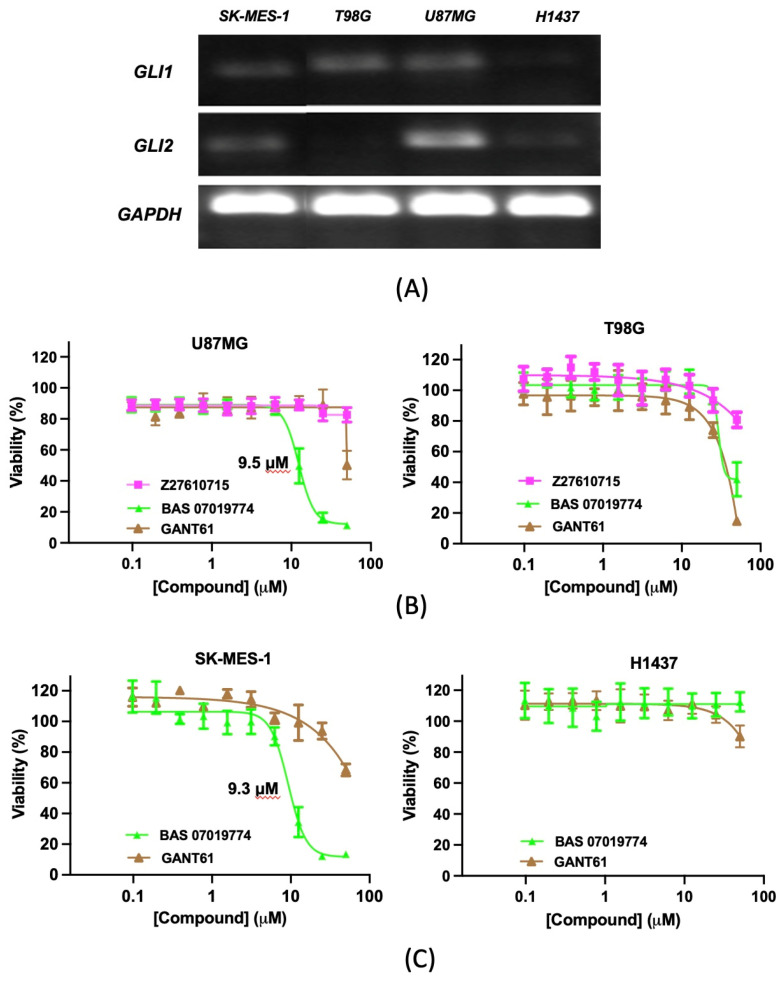
Effect of the selected GANT61 analogs on glioblastoma and lung cancer cell viability. (**A**) *GLI1* and *GLI2* mRNA expression levels were quantified in human glioblastoma and lung cancer cell lines (U87MG, T98G, SK-MES-1, and H1437) using TagMan qRT-PCR as described in Methods (see data in Supplemental Figure S5). PCR products were electrophoresed on 2% agarose gels and visualized by iBright 1500 Imaging System (Invitrogen, Carlsbad, CA, USA). The bands obtained for *GLI1*, *GLI2*, and *GAPDH* are at the predicted sizes of 80 bp, 88 bp, and 157 bp, respectively. Glioblastoma (**B**) and lung cancer (**C**) cell viability using MTT assay. Glioblastoma U87MG and T98G cells were seeded in 384-well plates at densities of 2500 and 1500 cells/well, respectively. SK-MES-1 and H1437 lung cancer cells were seeded in 384-well plates at densities of 1000 cells/well and 2000 cells/well, respectively. Cells were treated with the GANT61 analogs in dose–response at the indicated concentrations for 72 h, and their viability was assessed by MTT assay as described in Methods. Data are presented as the mean of % viability relative to that of cells treated with vehicle (0.1% DMSO). Dose–response curves were generated using non-linear regression, and IC_50_ values were determined using GraphPad Prism 9.

To evaluate the effect of the identified GANT61 analogs on cancer cell viability, we first assessed the expression of GLI1 and GLI2 in two human glioblastoma cell lines, U87MG and T98G, previously shown to be GANT61-sensitive and GLI1-dependent [71,72,73]. Consistent with previous reports [71,73], we observed that U87MG cells express both GLI1 and GLI2 and that T98G expresses predominantly GLI1 (Figure 5A and Supplemental Figure S5). The GLI1 mRNA expression was comparable in both U87MG and T98G (Figure 5A, Supplemental Figure S5). U87MG and T98G cells were incubated with GANT61 analogs for 72 h, and the cell viability was measured. Notably, BAS 07019774 showed significant effects on both U87MG and T98G cell viability (with IC_50_ values of 9.5 and 29.5 μM, respectively) (Figure 5B). Z27610715 exhibited a moderate reduction in T98G cell viability (a 19.2% reduction at 50 μM). Further, BAS 07019774 was more effective in reducing U87MG cell viability in comparison to GANT61, while their effects on TG98 cells were comparable (Figure 5B).

We further explored the effects of BAS 07019774 on two lung cancer cell lines, SK-MES-1 and H1437. SK-MES-1 has been shown to be GLI-dependent and GANT61-sensitive [74]. We found that the SK-MES-1 cells expressed both GLI1 and GLI2, which is consistent with a previous report [74], while the H1437 cells expressed minimal levels of GLI1 and GLI2 (Figure 5A and Supplemental Figure S5). Our data showed that BAS 07019774 was able to reduce the SK-MES-1 cell viability (with an IC_50_ value of 9.3 μM), whereas GANT61 had a modest effect (Figure 5C). Significantly, BAS 07019774 did not affect the viability of H1437 cells that have minimal GLI expression, while GANT61 had some effect on the viability of these cells at high concentrations (Figure 5C).

### 2.7. Predicted Binding Mode of BAS 07019774

**Figure 6:**
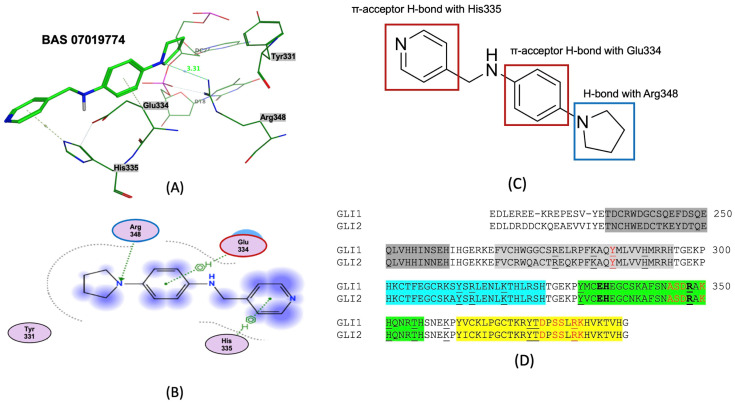
Predicted docking pose of BAS 07019774 to GLI1 zinc finger structure. BAS 07019774 was docked to the crystal structure of GLI1 five-zinc finger domain (PDB ID: 2GLI) using MOE. For clarity, residue numbering used in this figure corresponds to full-length GLI1 numbering (UNIPROT P08151). (**A**) The best docking pose predicted binding of BAS 07019774 to Arg348 (one hydrogen bond), Glu334 (π-acceptor H-bond), and His335(π-acceptor H-bond) residues within GLI1 ZF4. (**B**) Compounds and residues are shown in stick representation. Two-dimensional ligand interaction diagram of GLI1-ZF interactions with BAS 07019774. Ligand interactions were generated using MOE software (version MOE2022.02). (**C**) Chemical structure of BAS 07019774 annotated with GLI1-ZF interactions. (**D**) Amino acid sequence alignment of GLI1-ZF and GLI2-ZF domains is highlighted to show the following individual ZF sections: ZF1 (gray), ZF2 (light gray), ZF3 (blue), ZF4 (green), and ZF5 (yellow). Residues in bold (E, H, and R) correspond to Glu334, His335, and Arg348 and show where BAS 07019774 is predicted to bind. Residues in red are those that interact with DNA, and underlined residues are DNA phosphate contacts [75].

GANT61 was previously predicted to bind at the ZF region of GLI1 [41,45]. To understand how BAS 07019774 might interact with GLI1, we performed molecular docking simulations. Molecular docking was carried out using Molecular Operating Environment (MOE) and the human GLI1-ZF/DNA crystal structure (PDB ID: 2GLI) [75]. The 3D structures of BAS 07019774 and GANT61-D were generated using energy minimization in MOE. An evaluation of the 12 lowest-energy poses (visually, the number of key interactions; Supplemental Table S2) suggested pose 7 as the most favorable binding mode for BAS 07019774. In this predicted pose, BAS 07019774 interacts with ZF4, forming three interactions, one hydrogen (H)-bond with Arg348 and two π-acceptor H-bonds [76] with His335 and Glu334 (Figure 6A–C). These interacting residues are positioned near the GLI1-ZF DNA binding site (Figure 6D) and are also conserved between GLI1 and GLI2 proteins (Figure 6D). MOE predicted that GANT61-D also interacted in the same region of GLI1-ZF, forming two H-bonds, one with Arg348 and one with Glu334 (Supplemental Figure S6).

## 3. Discussion

As the hedgehog pathway has a major role in tumorigenesis, significant efforts have been made to develop drugs targeting this pathway [26,27], with several SMO inhibitors having been approved by the FDA [28,29,30]. Due to drug resistance to these SMO inhibitors [31], targeting elsewhere in the pathway, including downstream at the level of GLI, has been proposed [20,32,33,34,35,36,37,38]. Studies indicate that the inhibition of GLI may be more effective than SMO in blocking tumor growth in several cancer models [77,78,79]. GANT61 was one of first compounds shown to directly inhibit GLI1/2-mediated transcription [40] and was subsequently shown to bind directly to GLI1 [41]. However, the chemical instability and poor pharmacokinetic properties of GANT61 have prevented its development as an anti-cancer agent [20,45].

In this study, GANT61 and its active form GANT61-D were used as scaffolds to identify closely related compounds that may have improved potency and drug-like properties. From a set of fifty-two GANT61 analogs, we initially identified six with inhibitory effects (a low micromolar range) on the Hh pathway in cell-based assays. Five of these six GANT61 analogs were confirmed to be active in an orthogonal Hh-responsive *Gli* reporter cell-based assay. In C3HT101/2 cells, one of the compounds, BAS 07019774 (a *p*-phenylenediamine), significantly reduced *Gli1* mRNA at both 48 and 96 h. In contrast, Z27610715 was ineffective at 48 h but potent at 96 h, while GANT61 appeared to be more effective in inhibiting *Gli1* mRNA expression at 48 h rather than at 96 h (Figure 4), suggesting differing stabilities or solubilities amongst these compounds. The higher efficacy of GANT61 analogs in reducing *Gli1* expression compared to GANT61 at 96 h may be attributed, at least in part, to the reported low level of stability of GANT61 [45]. Further studies are required to assess the stability and the solubility of the selected GANT61 analogs (BAS 07019774 and Z27610715) compared to GANT61. Further, we endeavored to address some of the challenges outlined by Curran [80] in developing Hh pathway inhibitors, in particular, their potential off-target/cytotoxic effects at higher doses. We demonstrated that BAS 07019774 was effective at low micromolar concentrations in the Hh functional cellular assays without any significant cell cytotoxicity, indicating that the inhibition by BAS 07019774 was not simply due to cell killing independent of the Hh pathway. In contrast, high concentrations of GANT61 did appear to be cytotoxic for these cells. GANT61 has been reported to have differing cytotoxic effects on “normal” cell lines (see, for example, [81]).

In GLI-dependent cancer cell models, treatment with BAS 07019774 significantly reduced cell viability in both U87MG and T98G glioblastoma cell lines. Z27610715 showed a minimal effect on U87MG cells and a modest reduction in the viability of T98G cells. While GANT61 was less effective than BAS 07019774 in U87MG, they had comparable effects in T98G cells. Notably, BAS 07019774 was more effective in reducing the cell growth of U87MG, which expresses both GLI1 and GLI2, suggesting that BAS 07019774 potentially targets both GLI1 and GLI2. To provide more evidence for GLI targeting by this compound, BAS 07019774 was also found to be effective in reducing the viability of a GLI-dependent lung cancer cell line SK-MES-1 but was ineffective in a lung cancer line with minimal GLI expression (H1437). These findings suggest that the anti-cancer activity of BAS 07019774 could be linked to an ability to target GLI proteins.

Our evaluation of the molecular docking prediction of BAS 07019774 binding to the human GLI1-ZF domain [75] showed the best pose consisted of three interactions: one hydrogen (H)-bond with Arg348 and two π-acceptor H-bond interactions with the hydrogens of His335 and Glu334. These three residues are found within ZF4 of GLI1 (Figure 6). Notably, Arg348 is located between key residues in ZF4 (Figure 6D; Ala345, Ser346, Asp347, and Lys350), which are in contact with the conserved nine-base pair DNA binding site [3,75]. Furthermore, the crystal structure of GLI1-ZF showed that the Arg348 side chain forms an H-bond with the phosphodiester oxygen of the DNA backbone [75], suggesting that BAS 07019774 may disrupt GLI1-DNA binding. Interestingly, the residues interacting with BAS 07019774 (Arg348, His335, and Glu334 in GLI1) are conserved between GLI1 and GLI2 [41,75] (Figure 6D). This conservation suggests that BAS 07019774 might interact similarly with both GLI1 and GLI2. This could explain the observed higher effectiveness of BAS 07019774 in reducing the viability of cancer cell lines expressing both GLI1 and GLI2 (U87MG and SK-MES-1) compared to T98G cells, which primarily express GLI1. Our molecular docking predicts GANT61-D docking at a site on ZF4 close to or overlapping with that of BAS 07019774, with GANT61-D forming two H-bonds: one with Arg348 and one with Glu334. However, previous docking studies using AutoDock have the neutral form of GANT61-D docking between ZF2 and ZF3 with H-bond contacts with Glu250 and Glu298 [41,45,82,83]. The mutation of these residues reduced GANT61-GLI1 binding as assessed by surface plasmon resonance and reduced GANT61-mediated Hh-pathway inhibition [41]. In the study by Calcaterra et al. [45], it was proposed that the di-protonated form of GANT61-D would be the prevalent form under physiological conditions. Their docking studies had this di-protonated form of GANT61-D docking within ZF1 and ZF2 at a negatively charged surface with its interaction driven predominantly by electrostatic forces [45]. While the discrepancy between our data and those of the previous reports may be due to using different docking approaches, including, for example, the program utilized, initial pocket identification, grid density, and potential biases in scoring functions, there is precedent for some of the other identified GLI binders to potentially interact at different sites on GLI-ZF (see the discussion below on other identified GLI binders). Further, docking with transcription factors such as GLI1 can be challenging due to their flexibility, the absence of well-defined binding pockets, and their generally flat surfaces [39]. Further studies will be required to determine the functional significance of the residues on GLI1 identified by our study and predicted to be involved in BAS 07019774 binding.

**Figure 7:**
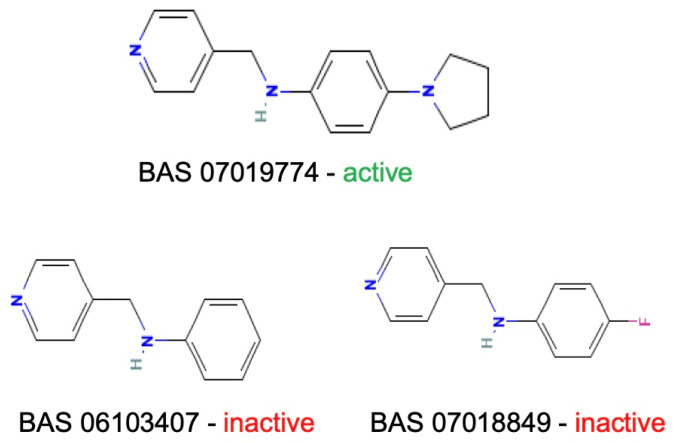
Preliminary SAR of BAS 07019774. Two compounds (BAS 06103407 and BAS 07018849) that have similar structures to BAS 07019774 were identified among the fifty-two analogs screened and found to be inactive. Both compounds maintain the *p*-diaminobenzene core but are missing the pyrrolidine ring, which makes an H-Bond with Arg348.

Based on the calculated chemical properties (Table 2), BAS 07019774 (pyridin-4-ylmethyl-(4-pyrrolidin-1-yl-phenyl)-amine) meets the criteria of Lipinski’s rule of five (mol. wt. < 500, LogP < 5, HBD < 5, HBA < 10) [84,85]. Further, its molecular weight is somewhat less than that for both GANT61 and GANT61-D. Due to the relatively low number of analogs we screened, exploring a structure–activity relationship (SAR) for BAS 07019774 is challenging. However, we did identify two closely related but inactive analogs (BAS 06103407 and BAS 07018849) that maintain the *p*-diaminobenzene core but are missing the pyrrolidine ring (Figure 7). In our docking study, this pyrrolidine ring makes an H-bond with Arg348. Further, the basicity of the pyrrolidine nitrogen at physiological pH is not very high, and it is likely to not be fully protonated. The low molecular weight for BAS 07019774 provides opportunities to add substituents to increase potency and improve physicochemical properties without exceeding 500 Da. BAS 07019774 has a “linear” arrangement of amine centers that nonetheless allows for medicinal chemistry exploration to optimize its properties. The *p*-diaminobenzene core could be maintained with substitutions at any position or the addition of other heteroatoms to the rings in order to find analogs. An alternative would be to preserve the pyrrolidino-phenylenediemaine structure and interrogate the SAR around that moiety. The pyrrolidine ring of BAS 07019774 could also be opened or modified without negatively impacting any of the interactions suggested in this work.

**Figure 8:**
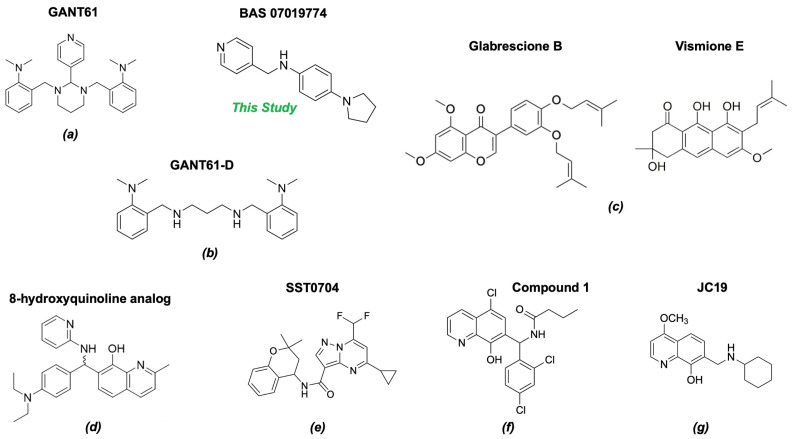
Chemical structures of reported direct GLI1 binding inhibitors. Structures were drawn in “ACS 1996” format with ChemDraw exchange file type (.cdx) or generated from SMILES files downloaded from PubChem. Compounds from the following references: (**a**) [40], (**b**) [45], (**c**) [39], (**d**) [82], (**e**) [90], (**f**) [91] and (**g**) [92].

Only GANT61 [41,45], the natural isoflavone Glabrescione B (GlaB) (Figure 8) [39], and arsenic trioxide [49] have been shown to directly bind GLI1. GlaB was identified from a virtual screening of >800 compounds based on mutagenesis studies identifying residues involved at the GLI1-DNA binding site [39]. The docking studies predicted GlaB binding to a groove between ZF4 and ZF5 with interactions to Lys340 and Lys350, interfering with the interaction of GLI1 with its target DNA [39]. GlaB derivatives have been designed that can target both GLI1 and SMO [86,87,88]. The chemical structures of GANT61 [82,89] and GlaB [90] have been used as scaffolds for virtual screening to identity other compounds that act as GLI1 inhibitors. In particular, GlaB and vismione E (Figure 8), which were previously discovered by Infante et al. [39], were used to generate a multi-feature pharmacophore that identified thiophene and pyrazolo-pyrimidine compounds that were predicted to dock in the same binding pocket as GlaB [90]. One of the pyrazolo-pyrimidine compounds (SST0704, Figure 8) had predicted docking interactions with ZF4 [90]. This same five-feature pharmacophore was subsequently used to identify several 8-hydoxyquinoline derivatives [91] that had a similar scaffold to another 8-hydoxyquinoline analog (Figure 8) [82], which was identified by virtual screening based on biased docking focused on the GANT61 binding site within ZF2 and ZF3. Interestingly, in the same study [91], a compound that matched the five-feature model (compound **1**, Figure 8) could be docked in two alternative best-docking poses: one at ZF4, as predicted for GlaB, and the other between ZF1 and ZF3, as predicted for GANT61. Further, they showed that some of the derivatives they identified could be docked at ZF4, while others also had an alternative best-scoring docking pose with the putative GANT61 binding site. Docking studies of a new 8-hydoxyquinoline derivative JC19 (Figure 8) [92] using putative binding sites at ZFs 1, 3, and 4 showed interactions at ZF4/5 similar to those hypothesized previously for other derivatives [90], as well as GlaB [39]. JC19 inhibits the formation of the GLI1-DNA complex by making predicted interactions with residues in ZF4 and ZF5, in particular, His351 and His356 [92]. These studies highlight some of the challenges with docking simulations for GLI1 and suggest that there are some pharmacophores that can be docked at both sites (“GlaB” and “GANT61”). Indeed, a recent study shows GANT61 docking with other Hh pathway components’ SMO and SUFU [93].

While our unbiased docking for BAS 07019774 on GLI1 ZF predicted a significant cluster of the top-ranked poses at ZF4, with a predicted best pose fitting with our preliminary SAR data, we have also found that using biased docking at the GANT61 site (with a grid at Glu250/Glu298 (full-length GLI1 numbering)) can generate docking poses that have some reasonable interactions with BAS 07019774 (see an example in Supplemental Table S3). However, none of the top 15 predicted poses from this biased docking study have interactions with the pyrrolidine ring of BAS 07019774 and thus do not fit with our SAR. While some of these other GLI-ZF binding compounds (Figure 8) could bind in a similar fashion to BAS 07019774, there is not much chemical structural overlap. However, the 8-hydoxyquinoline analog ((*d*) in Figure 8) [82] is an exception that can be mapped almost directly to BAS 07019774. Further exploration around both scaffolds is necessary to establish any correlations and provide valuable insights.

## 4. Materials and Methods

### 4.1. Cell Lines, Reagents, and Compounds

Cell lines C3H10T1/2 (Clone 8), U87MG, T98G, SK-MES-1, and H1437 were obtained from American Type Culture Collection (ATCC, Manassas, VA, USA). The *Gli*-luciferase reporter NIH3T3 cell line was obtained from BPS Bioscience (San Diego, CA, USA). Cells were cultured following the manufacturer’s instructions. In the case of C3H10T1/2, they were optimized for high-throughput screening (HTS) using DMEM. All experiments were conducted using cells between passage numbers 3 and 8. GANT61 was purchased from TOCRIS (Minneapolis, MN, USA). KAAD-cyclopamine (KAAD-cyc) and SAG were obtained from MilliporeSigma (Burlington, MA, USA). Purmorphamine was purchased from Cayman Chemical (Ann Arbor, MI, USA). Hoechst-33342 dye, SYPR Safe DNA gel stain, and agarose (genetic technology grade) were purchased from Themo Fisher Scientific (Waltham, MA, USA). GANT61 analogs were purchased from Enamine (Kyiv, Ukraine) or Asinex Corp (Winston-Salem, NC, USA). All compounds were purchased as powders, dissolved in 100% DMSO to obtain 10 mM and 50 mM stock solutions, and stored at −20 °C. 

### 4.2. C3H10T1/2 Hedgehog-Responsive High-Throughput Cell-Based Assay 

To assess effects on Hh activity, we used the murine embryonic fibroblast cell line C3H10T1/2 [64]. These cells have been previously demonstrated by our group and others to be responsive to Hh signaling [63,65]. C3H10T1/2 cells were cultured in Dulbecco’s Modified Eagle’s Medium (DMEM) supplemented with 10% FBS and plated in black, clear-bottomed 384-well plates (Thermo Fisher Scientific, Waltham, MA, USA) with 1500 cells/well using a MultiFlo cell dispenser (Agilent BioTek, Winooski, VT, USA). After 24 h, cells were treatedw with Smoothened agonist (SAG) [94,95] at its half-maximal effective concentration (EC_50_ = 30 nM) to induce Hh activity. GANT61 analogs in 100% DMSO were then added to the cell plates in a 10-point 2-fold dose–response format, and normalization performed using a D300 digital dispenser (HP). Cells were incubated for 5 days and lysed, and alkaline phosphatase (AP) activity was measured to assess Hh activity. The AP fluorescent substrate [96] (AttoPhos^®^, Promega, Madison, WI, USA) was added to each well, plates were incubated in the dark for 30 min, and fluorescence measured at Ex/Em 435/555 nm using a CLARIOstar plate reader (BMG Labtech, Cary, NC, USA). This automated 384-well C3H10T1/2 assay with fluorescence readout was adapted from our previous 96-well colorimetric version [97,98]. For some C3H10T1/2 cell experiments, a 384-well colorimetric readout assay (pNPP substrate, 405 nm absorbance, clear plates) was used. Purmorphamine (PUR) [67,68], an alternative SMO agonist, was used in some experiments to activate Hh activity in C3H10T1/2 cells (PUR used at its EC_50_ value of 500 nM, determined by dose–response). AP activity was normalized to SAG-induced or PUR-induced cells (maximum signal control, columns 1 and 2 in each 384-well assay plate). As a minimum signal control, cells were treated with 30 nM of SAG plus 40 nM (IC_90_) of known Hh inhibitor KAAD-cyclopamine (KAAD-cyc) [69] (columns 23 and 24 in each 384-well assay plate). IC_50_ values were calculated using variable slope (four-parameter dose response) in Prism 9 GraphPad software.

### 4.3. Gli-Luciferase NIH3T3 Reporter Assay 

*Gli* reporter NIH3T3 cells (BPS Bioscience, catalog# 60409), a stable cell line that contains firefly luciferase gene under the control of Gli-responsive elements, were used to measure Hh pathway activity. Cells were grown in 96-well plates at a density of 25,000 cells/well for 24 h in Thaw Medium 5 (BPS Bioscience). After 24 h, the medium was replaced with fresh assay media (100 μL) using a Biomek^®^NX (Brea, CA, USA) automated liquid handler. To activate the Hh pathway, cells were treated with SAG at its half-maximal effective concentration (EC_50_ = 26 nM). To assess inhibition, the GANT61 analogs were added (serial dilutions of 0.098 to 50 μM) using a D300 system, and cells were then incubated at 37 °C for 30 h. Firefly luciferase activity was measured using the One-Step Luciferase Assay, according to vendor’s protocol (BPS Bioscience, catalog # 60690-1). Briefly, One-Step luciferase reagent (100 μL) was added to each well and incubated by shaking for 20 min at room temperature, followed by luminescence detection using a GloMax plate reader (Promega). To account for background luminescence, cell-free control wells were included. The background-subtracted luminescence of cells stimulated with SAG alone was set as 100% luminescence for calculating percent inhibition of the test compounds. The inhibition curves were plotted using GraphPad Prism 9 to determine IC_50_ values. Each assay was repeated at least three times.

### 4.4. Cytotoxicity Assay

Cytotoxicity of compounds was evaluated in C3H10T1/2 and *Gli* reporter NIH3T3 cell lines. Cells were plated and treated with SAG (at its EC_50_ concentration), and compounds were added in dose–response as above. For C3H10T1/2 cells, Hoechst dye 33342 (10 µg/mL) was added to each well 5 d after treatment. For *Gli* reporter NIH3T3 cells, Hoechst dye was added 24 h after treatment. Cells were incubated with the Hoechst dye for 45 min at 37 °C. Following incubation, cells were washed with (50 μL) PBS (phosphate-buffered saline), fixed with formalin (20 μL), and incubated in the dark at room temperature for 15 min. After fixing the cells, formalin was removed, and 50 μL of PBS added. High-content imaging was performed using a Cellinsight NXT system (Thermo Fisher) at an excitation wavelength of 386 nm. Data were analyzed using GraphPad Prism 9.

### 4.5. qRT-PCR Assay to Measure GLI mRNA Expression

*Gli1* mRNA expression in murine C3H10T1/2 was quantitatively assessed by qRT-PCR, as previously described [96]. Briefly, C3H10T1/2 cells were seeded at 60,000 cells/well in 12-well plates in DMEM/10% FBS. After 24 h, cells were treated with SAG (EC_50_ = 30 nM) in the presence of GANT61 or GANT61 analogs (10 μM). Cells were harvested 48- and 96-h post-treatment (with three replicate treatments per group). *Gli1* mRNA induction by SAG over a time course (0, 12, 24, 48, 72, 96, and 120 h) was assessed in a separate experiment. Untreated cells were used as a control. Total RNA was isolated using RNeasy Mini Plus Kit (Qiagen, Germantown, MD, USA). RNA (1 μg) was reverse-transcribed into cDNA using an iScript cDNA Synthesis Kit (Bio-RAD, Hercules, CA, USA). qRT-PCR was performed to quantify mRNA using TaqMan assay. Probes specific for murine *Gli1* (Mm00494654_m1) and murine *β-actin* (Mmo2619580_g1) were obtained from Applied Biosystems (Thermo Fisher Scientific). Target sequences were amplified at 95 °C for 10 min followed by 40 cycles at 95 °C for 15 s and 60 °C for 1 min. Each experiment was performed in triplicate on a QuantStudio 6 flex (Thermo Fisher Scientific). For normalization, *β-actin* was utilized as an endogenous control, and the fold change was determined using the 2^−△△Ct^ method.

*GLI1* and *GLI2* mRNA expression were quantified in human cancer cell lines (U87MG, T98G, SK-MES-1 and H1437) using TaqMan qRT-PCR as described above. Probes specific for human *GLI1* (Hs00171790_m1), *GLI2* (Hs01122187_m1), and *GAPDH* (Hs02786624_g1) were used to amplify the target sequences. The housekeeping gene GAPDH was used for normalization and to determine fold change using the 2^−△△Ct^ method. For gel electrophoresis, PCR products were run on agarose gels (2%) for 90 min at a constant 150 V. Gels were visualized with Syber Safe DNA stain and imaged using an iBright 1500 Imaging System (Invitrogen).

### 4.6. Cancer Cell Viability Assay

Glioma cell lines T98G and U87MG were plated in 384-well (clear, flat-bottomed) plates in EMEM/10% FBS (50 µL) at densities of 1500 and 2500 cells/well, respectively. Lung cancer cell lines SK-MES-1 and H1437 were plated at cell densities of 1000 cells/well in 50 µL EMEM and 2000 cells/well in 50 µL RPMI-1640, respectively. After 24 h, cells were treated with selected GANT61 analog compounds using a 10-point, 2-fold dose–response curve (0.098–50 µM) and incubated at 37 °C for 72 h. To assess cell viability, MTT was added to each well at a final concentration of 0.5 mg/mL using a Biomek^®^NX (Brea, CA, USA) automated liquid handler followed by incubation for 4 h at 37 °C. Media and MTT were then removed, DMSO (40 µL) was added to each well to dissolve the formed formazan crystals, and they were incubated for 1 h. Absorbance was then measured at 550 nm using a SpectraMax plate reader (Molecular Devices, San Jose, CA, USA). Cells treated with 0.1% DMSO were used as a control. The absorbance values from compound-treated wells were normalized to the control. The normalized absorbance values were used to calculate the percentage of viable cells remaining after treatment with the compounds. Data were analyzed using Prism 9 (GraphPad, Boston, MA, USA).

### 4.7. Molecular Docking

The crystallographic structure of human GLI1-ZF/DNA complex [75] (PDB ID: 2GLI) was utilized as a rigid structure to perform molecular docking using the Molecular Operating Environment (Molecular Operating Environment (MOE, version MOE2022.02), Chemical Computing Group ULC, Montreal, QC, Canada) to determine the binding mode of the identified GANT61 analogs and GANT61-D. All solvent molecules were removed from the structure prior to docking. Unbiased molecular docking simulations were carried out using MOE’s default parameters. The chemical structure of BAS 07019774 was obtained from eMolecules (emolecules.com, accessed on 11 August 2023), and GANT61-D structure was as published previously [45]. Three-dimensional structure poses were generated in MOE software by energy minimization. The top-ranking poses were evaluated by comparing the docking scores (predicted binding affinity) and the number of key interactions formed.

### 4.8. Statistical Analysis

All cell culture and in vitro experiments were independently repeated at least three times under the same conditions. All assays were performed in triplicate. All data are presented as the mean ± SD. One-way analysis of variance (ANOVA) followed by Tukey’s HSD multiple comparisons, *t*-test, and Mann–Whitney test were used to evaluate the significant differences between treatments. GraphPad Prism 9 software (San Diego, CA, USA) was used for statistical analysis. A value of *p* < 0.05 was considered statistically significant.

## 5. Conclusions

In conclusion, this study aimed to identify more effective Hh pathway inhibitors that target GLI. In contrast to virtual screening approaches, we utilized lab-based high-throughput technologies to successfully screen a compound library of fifty-two analogs that we identified based on the chemical structures of GANT61 and GANT61-D. Five promising analogs inhibited Hh activity in two independent cell-based assays without significant cytotoxicity. Notably, one analog, BAS 07019774, effectively decreased *Gli1* mRNA expression in an Hh-responsive cell line model and reduced cell viability in GLI-dependent cancer cell lines. Future studies will be required to evaluate the in vivo efficacy and pharmacokinetic properties of BAS 07019774. The data presented in this study provide a valuable foundation to facilitate medicinal chemistry studies to elucidate the full potential of these analogs as novel Hh pathway inhibitors.

## Figures and Tables

**Table 1:** Activity testing in C3H10T1/2 and Gli-luciferase NIH3T3 cell-based assays for compounds identified from primary screening.

Compound	Chemical Structure	C3H10T1/2 (Flu AP, IC_50_ μM)	Gli-NIH3T3 (luciferase, IC_50_ μM)	C3H10T1/2 (Abs AP, IC_50_ μM)	C3H10T1/2 (PUR; Flu AP, IC_50_ μM)
Z27613695	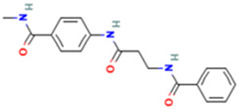	<20% *^a^*	<20%	<20%	<20%
Z27610715	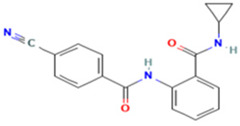	1.1	6.7	<20%	8.1
BAS 06844821	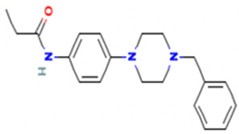	<20%	~40%	<20%	<20%
BAS 09681156	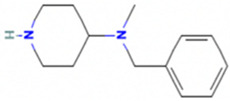	<20%	3.8	~30%	~40%
BAS 07019774	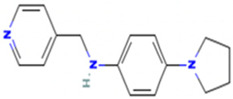	5.5	11.2	3.3	6.5
BAS 06348344	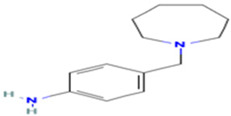	<20%	0.9	<20%	<20%
GANT61	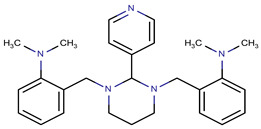	10.5	1.9	3.7	2.5

*^a^* For values given as %, this refers to % inhibition at highest dose tested.

**Table 2:** Calculated properties for BAS 07019774 and GANT61.

Compound	Mol. wt. (g/mol)	cLogP	LogS	PSA	RB	HBD	HBA
GANT61	429.6	4.9	−5.9	25	7	0	5
GANT61-D	340.5	3.9	−4.3	31	10	2	4
BAS 07019774	253.3	1.9	−3.7	28	4	1	3

Abbreviations: cLogP, calculated lipophilicity of partition coefficient; LogS, calculated aqueous solubility; PSA, polar surface area; RB, number of rotational bonds; HBD, number of hydrogen bond donors; HBA, number of hydrogen bond acceptors. Properties calculated in ChemDraw 23 (Revvity).

## Data Availability

The raw data supporting the conclusions of this article will be made available by the authors upon request.

## Supplementary Materials

The following supporting information can be downloaded at: https://www.mdpi.com/article/10.3390/molecules29133095/s1, Figure S1: Activity of Hh pathway agonist and antagonist controls in Hh-responsive cell lines C3H10T1/2 and *Gli*-reporter NIH3T3 cellular assays.; Figure S2: Screening of GANT61 analog set in C3H10T1/2 cells using AP absorbance assay.; Figure S3: Effects of selected GANT61 analogs in C3H10T1/2 stimulated by different Smo agonists.; Figure S4: Time course of *Gli1* mRNA expression in C3H10T1/2 cells.; Figure S5: Quantification of *GLI1* and *GLI2* mRNA expression in tumor cells.; Figure S6: Molecular docking prediction for the binding mode of GANT61 to GLI1.; Table S1: Compound ID and chemical structures for the GANT61 analog compound set used in this study.; Table S2: Top 12 predicted poses for unbiased docking of BAS 07019774 to GLI1-ZF.; Table S3: Table of Top 15 predicted poses for biased docking of BAS 07019774 to GLI1-ZF at Glu119/Glu167.
