# Supplementary material for: Identification of Novel GANT61 Analogs with Activity in Hedgehog Functional Assays and GLI1-Dependent Cancer Cells
Source: Molecules. 2024 Jun 28;29(13):3095. doi: 10.3390/molecules29133095 (PMC11243198; doi:10.3390/molecules29133095)
Supplement: Supplementary file 1 [file molecules-29-03095-s001.zip › molecules-3062436-supplementary.pdf]

Identification of Novel GANT61 Analogs with Activity in Hedgehog Functional Assays and Gli1-Dependent Cancer Cells

Dina Abu Rabe [1,*], Lhoucine Chdid [2], David R. Lamson [2], Christopher P. Laudeman [2], Michael Tarpley [2], Naglaa Elsayed [2], Ginger Smith [2], Weifan Zheng [2,3], Maria S. Dixon [2,*], Kevin P. Williams [2,3,*]

[1] INBS PhD Program, [2] Biomanufacturing Research Institute and Technology Enterprise,

[3] Department of Pharmaceutical Sciences, North Carolina Central University, Durham, NC 27707, USA

[*]Authors to whom correspondence should be addressed.

Table of Contents for Supplemental Material

Figure S1: Activity of Hh pathway agonist and antagonist controls in Hh-responsive cell lines C3H10T1/2 and *Gli*-reporter NIH3T3 cellular assays.

Figure S2: Screening of GANT61 analog set in C3H10T1/2 cells using AP absorbance assay.

Figure S3: Effects of selected GANT61 analogs in C3H10T1/2 stimulated by different Smo agonists.

Figure S4: Time course of *Gli1* mRNA expression in C3H10T1/2 cells.

Figure S5: Quantification of *GLI1* and *GLI2* mRNA expression in tumor cells.

Figure S6: Molecular docking prediction for the binding mode of GANT61 to GLI1.

Table S1: Compound ID and chemical structures for the GANT61 analog compound set used in this study.

Table S2: Top 12 predicted poses for unbiased docking of BAS 07019774 to GLI1-ZF.

Table S3: Table of Top 15 predicted poses for biased docking of BAS 07019774 to GLI1-ZF at Glu119/Glu167.

Figure S1

(A)

C3H10T1/2 AP Fluorescence Assay

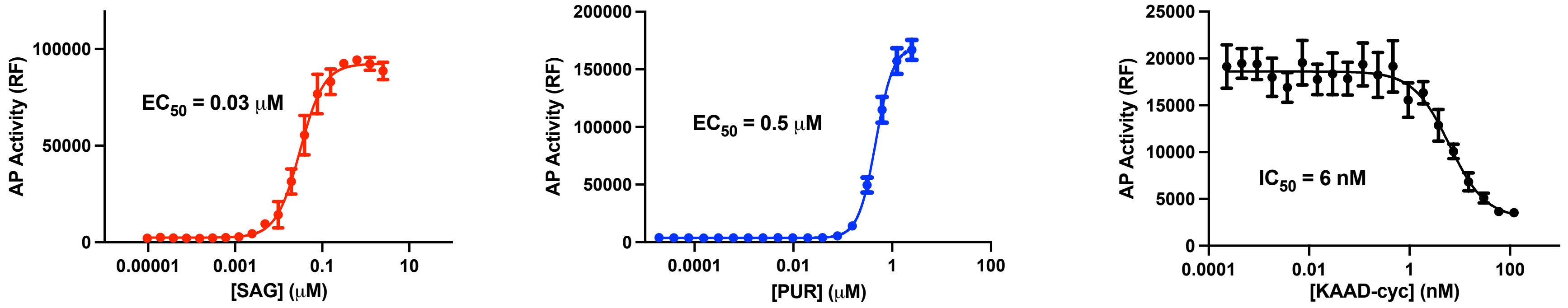

(B)

Gli-Reporter NIH3T3 Luciferase Assay

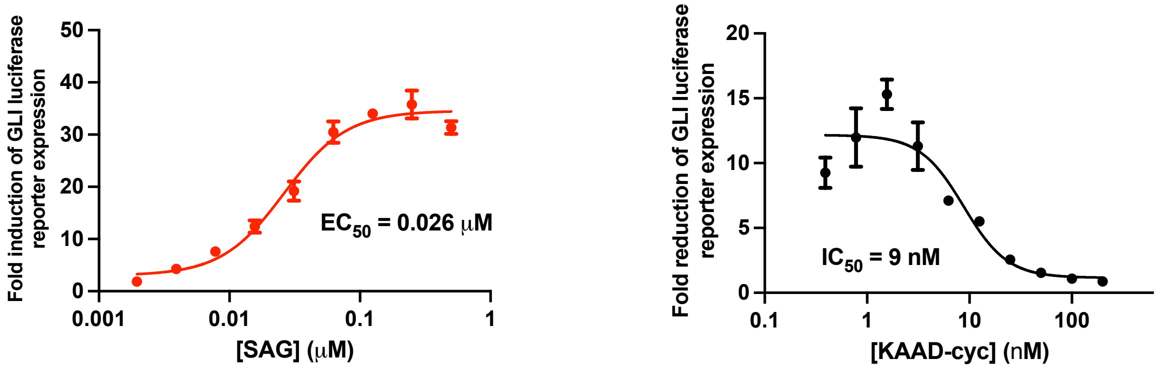

Figure S2

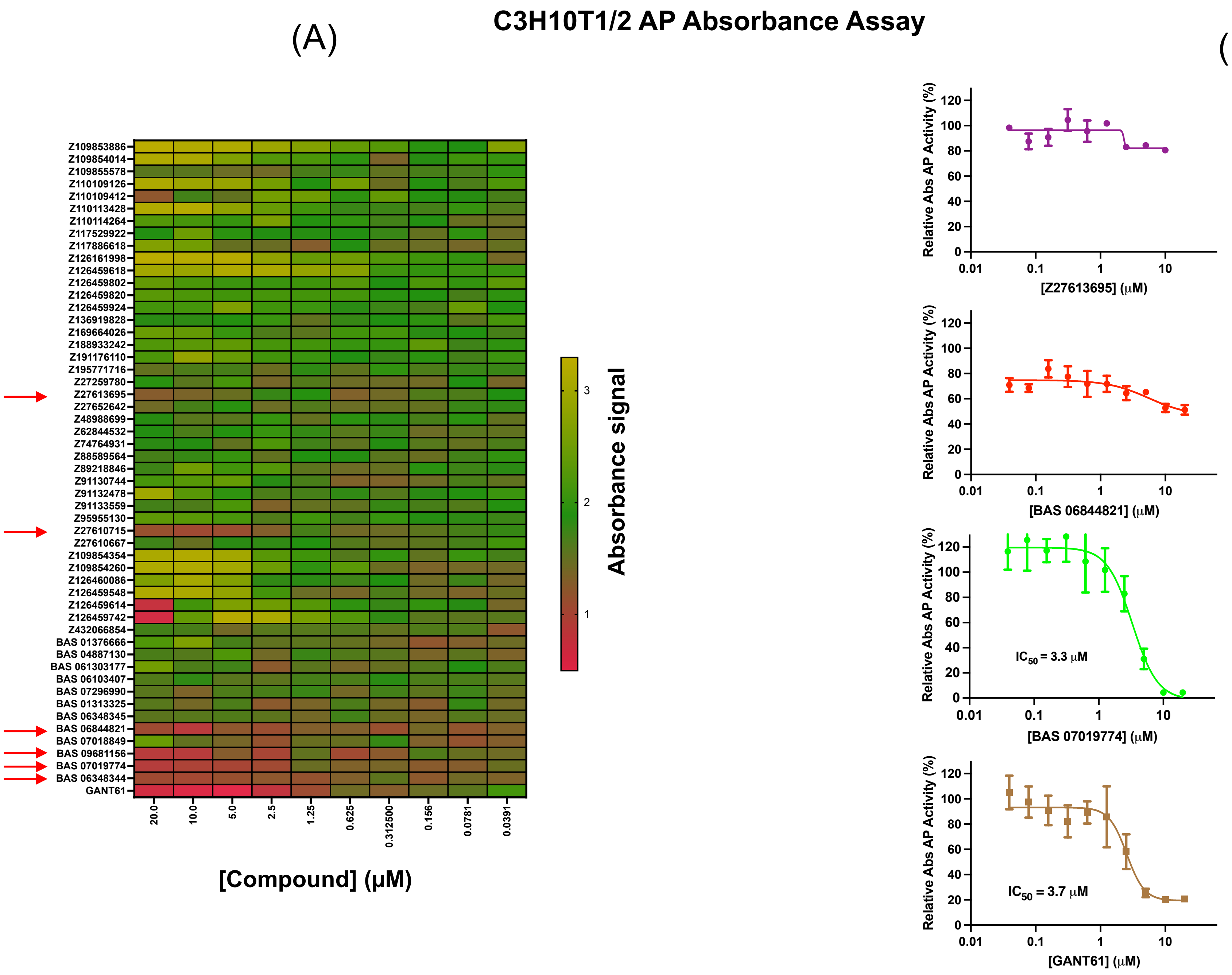
C3H10T1/2 AP Absorbance Assay
(A)
(B)
Absorbance signal
[Compound] (μM)
IC50 = 3.3 μM
IC50 = 3.7 μM
[Z27613695] (μM)
[Z27610715] (μM)
[BAS 06844821] (μM)
[BAS 09681156] (μM)
[BAS 07019774] (μM)
[BAS 06348344] (μM)
[GANT61] (μM)
Relative Abs AP Activity (%)

Figure S3

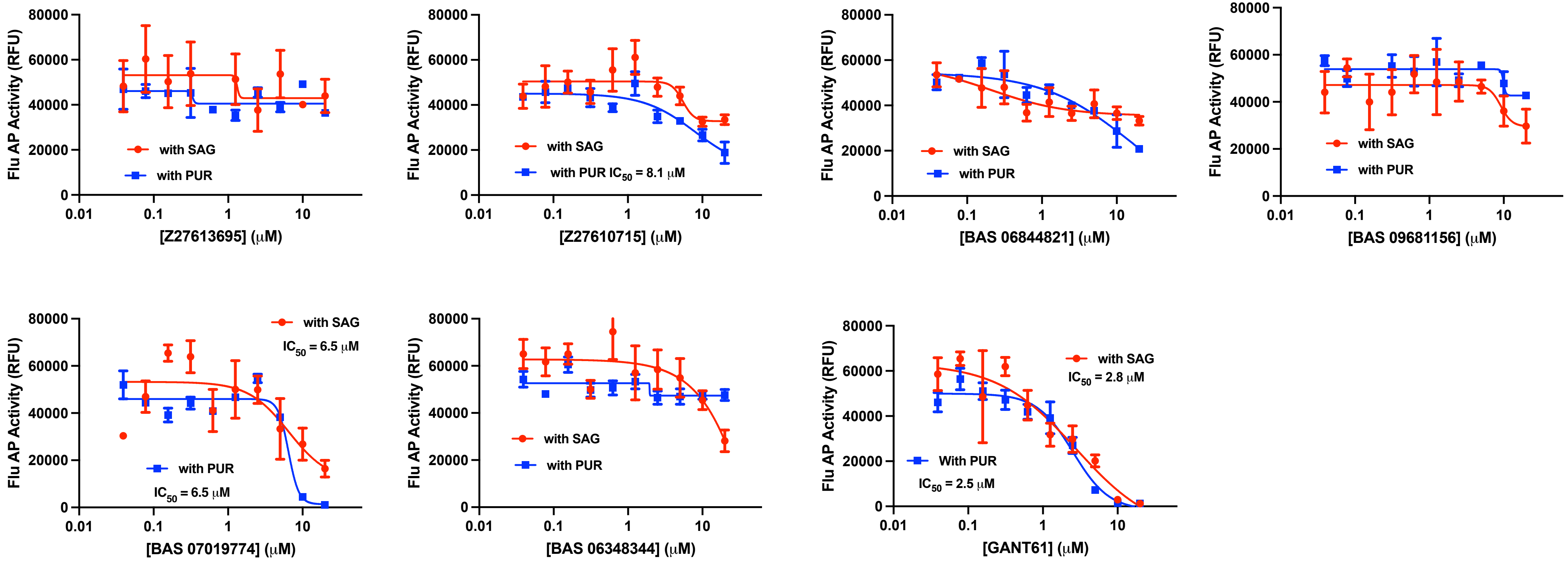

Flu AP Activity (RFU)
with SAG
with PUR
[Z27613695] (μM)
with PUR IC50 = 8.1 μM
[Z27610715] (μM)
[BAS 06844821] (μM)
[BAS 09681156] (μM)
IC50 = 6.5 μM
[BAS 07019774] (μM)
[BAS 06348344] (μM)
IC50 = 2.8 μM
With PUR
IC50 = 2.5 μM
[GANT61] (μM)

Figure S4

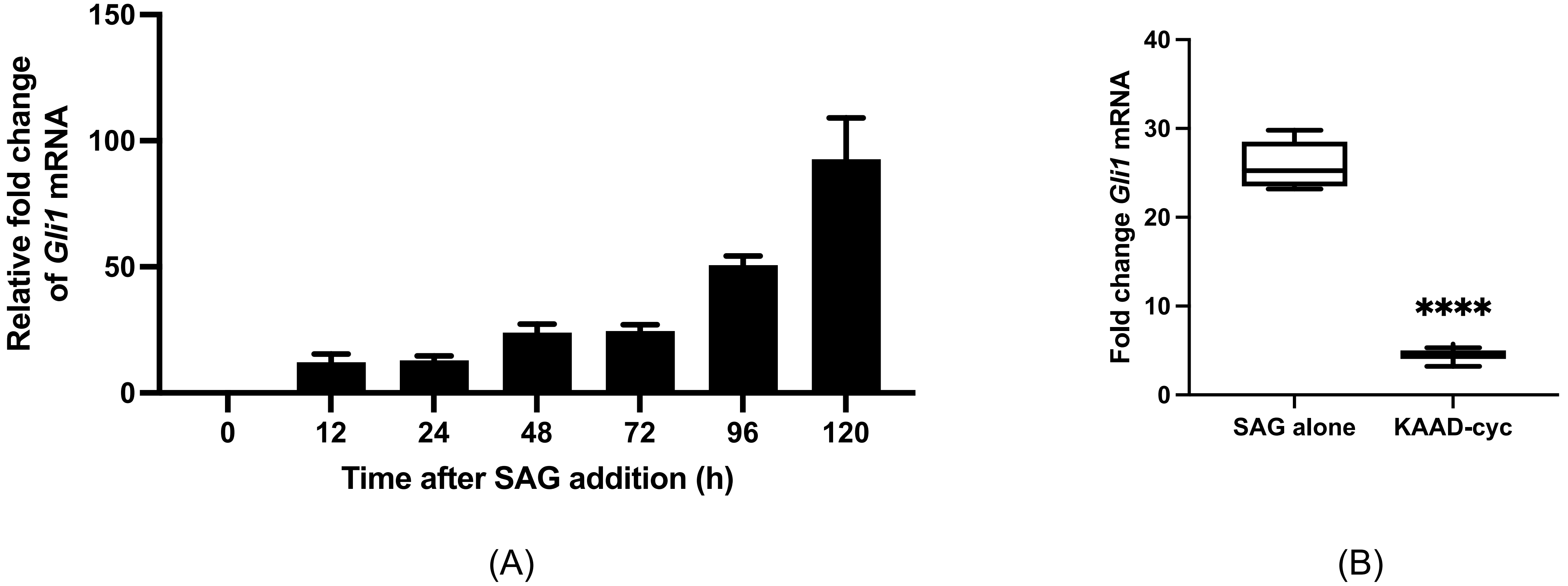
150
100
50
0
Relative fold change of Gli1 mRNA
0
12
24
48
72
96
120
Time after SAG addition (h)
(A)
40
30
20
10
0
Fold change Gli1 mRNA
****
SAG alone
KAAD-cyc
(B)

Figure S5

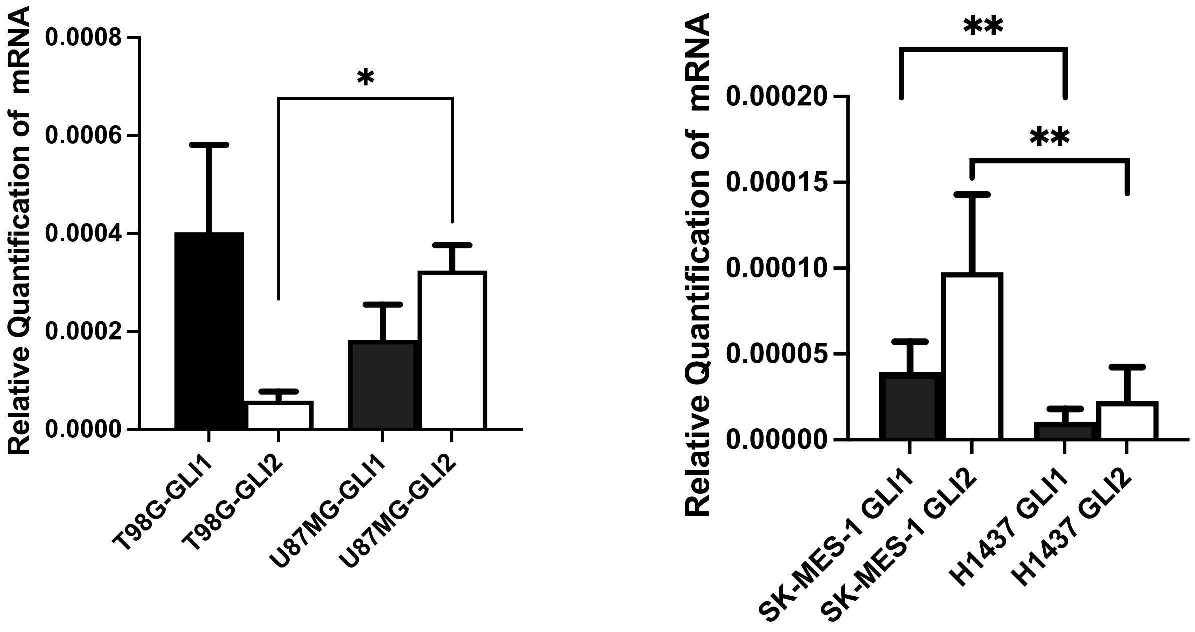

Relative Quantification of mRNA
0.0008
0.0006
0.0004
0.0002
0.0000
*
T98G-GLI1
T98G-GLI2
U87MG-GLI1
U87MG-GLI2
Relative Quantification of mRNA
0.00020
0.00015
0.00010
0.00005
0.00000
**
**
SK-MES-1 GLI1
SK-MES-1 GLI2
H1437 GLI1
H1437 GLI2

(A) (B)

Figure S6

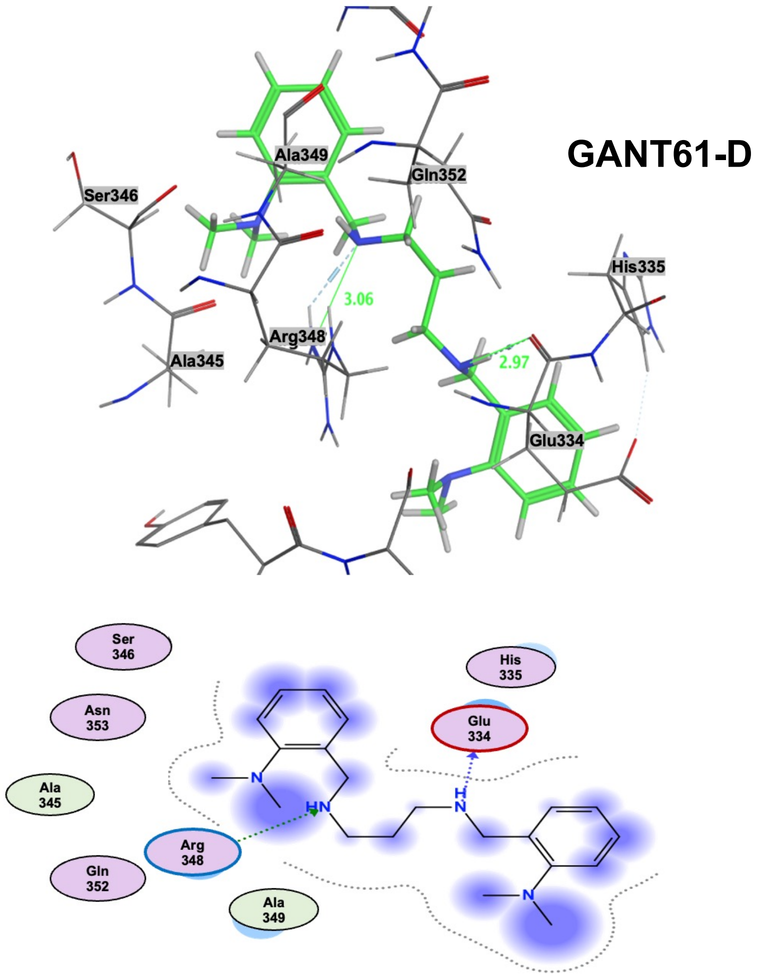
GANT61-D
Ala349
Gln352
Ser346
His335
3.06
Arg348
Ala345
2.97
Glu334
Ser 346
His 335
Asn 353
Glu 334
Ala 345
HN
H N
N
N
Arg 348
Gln 352
Ala 349

Figure S1: Activity of Hh pathway agonist and antagonist controls in Hh-responsive cell lines C3H10T1/2 and *Gli*-reporter NIH3T3 cellular assays. (**A**) For agonist dose response curves, SAG or PUR were added to C3H10T1/2 cells at the indicated concentrations and cells incubated for a further 5 days. For antagonist dose response curve, C3H10T1/2 cells were stimulated with SAG at its EC_{50} value (30 nM) and KAAD-cyc added at the indicated concentrations. AP activity was measured using a fluorescent substrate as described in Methods. All experiments conducted as a minimum of three independent experiments. (B) For agonist dose response curves, SAG was added to *Gli*-reporter NIH3T3 cells at the indicated concentrations and cells incubated for a further 30 h. For antagonist dose response curve, *Gli*-reporter NIH3T3 cells were stimulated with SAG at its EC_{50} value (26 nM) and KAAD-cyc added at the indicated concentrations. One-step luciferase assay was performed as described in Methods. Data are presented as the mean of fold change of GLI luciferase reporter expression ± SD of three replicates. EC_{50} and IC_{50} values were determined by non-linear regression in GraphPad Prism 9.

Figure S2: Screening of GANT61 analog set in C3H10T1/2 cells using AP absorbance assay. (**A**) Representative heat map profile for Hh-pathway inhibitory effects of the GANT61 analog set in C3H10T1/2 cells. C3H10T1/2 cells were stimulated with SAG at its EC_{50} value (30 nM), GANT61 analogs added in dose response at the indicated concentrations and after 5 days, AP activity was measured at 405 nm using *p*NPP substrate. Each row represents dose response data for a single GANT61 analog. The heat map key indicates red for maximum inhibition through to pale green for minimal inhibition. Red arrows indicate GANT61 analogs selected for further study. (**B**) Dose response curves for selected GANT61 analogs in C3H10T1/2 cells using colorimetric readout as in A. Data were normalized to SAG-stimulated cells and displayed as the mean of % activity (n =

3 independent experiments). Dose response curves were generated using non-linear regression and IC_{50} values determined in GraphPad Prism 9.

Figure S3: Effects of selected GANT61 analogs in C3H10T1/2 stimulated by different Smo agonists. C3H10T1/2 cells were treated with a fixed concentration of either SAG (30 nM) or PUR (500 nM) and GANT61 analogs added at the indicated concentrations. After 5 days, AP activity was measured using the fluorescence substrate and readout. Dose response curves were generated using non-linear regression and IC_{50} values determined in GraphPad Prism 9.

Figure S4: Time course of *Gli1* mRNA expression in C3H10T1/2 cells. (**A**) SAG induction of *Gli1* mRNA expression at different time points. C3H10T1/2 cells were treated with SAG at its EC_{50} (30 nM), cells collected at the indicated time points for RNA extraction and cDNA synthesis. *Gli1* mRNA expression was determined using TaqMan qRT-PCR. (**B**) KAAD-cyc inhibition of SAG-induced *Gli1* mRNA at 48 h in C3H10T1/2 cells. Cells were treated with 30 nM SAG with and without KAAD-cyc at its IC_{90} value (40 nM). Data was normalized to the housekeeping gene β-actin and displayed as the mean of fold change of *Gli1* mRNA expression ± SD of three independent experiments. Two-tailed t-test and Mann-Whitney test represent SAG + KAAD-cyc significantly different (****p-value <0.0001) from SAG alone.

Figure S5: Quantification of *GLI1* and *GLI2* mRNA expression in tumor cells. (**A**) Glioblastoma U87MG and T98G. (**B**) Lung cancer SK-MES-1 and H1437. *GLI1* and *GLI2* mRNA was quantified using TaqMan RT-PCR. The housekeeping gene *GAPDH* was used for normalization and to determine fold change using the $2^{-\Delta\Delta Ct}$ method. Data presented as the mean of the relative

quantification of mRNA ± SD of three replicates.–One-way ANOVA and Tukey's multiple comparisons show significantly different (* and **p-value < 0.05) expression of GLI1 and GLI2 mRNA between cell lines.

Figure S6: Molecular docking to predict the binding mode of GANT61 to GLI1 using the crystal structure of the five-zinc finger domain (PDB ID: 2GLI1). Best-ranked docking pose predicted binding of GANT61 to GLI1-ZF domain at amino acid residues Glu334 and Arg348. Ligand interactions were generated in MOE software.

Supplemental Table S1. Compound ID and chemical structures for the GANT61 analog compounds used in this study.

Compound Vendor ID	Compound Structure
**Z109853886**	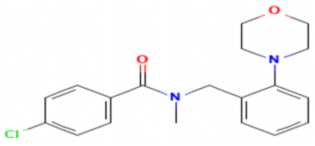
**Z109854014**	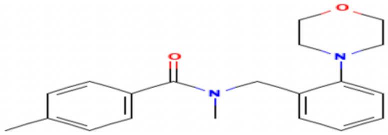
**Z109855578**	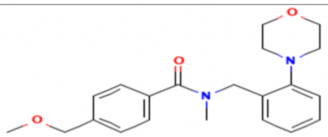
**Z110109126**	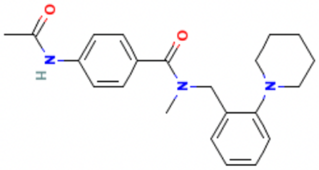
**Z110109412**	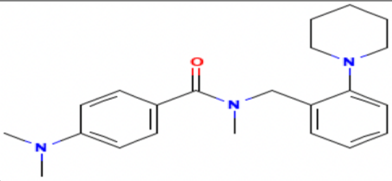
**Z110113428**	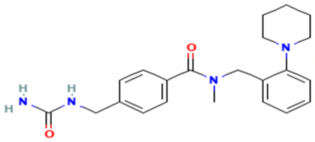
**Z110114264**	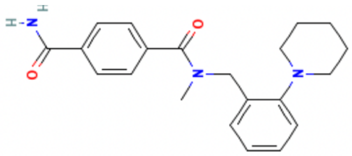
**Z117529922**	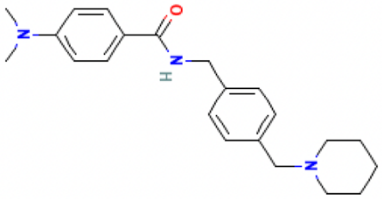

**Z117886618**	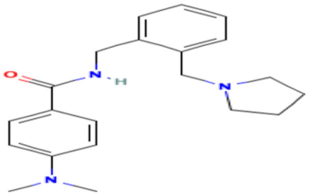
**Z126161998**	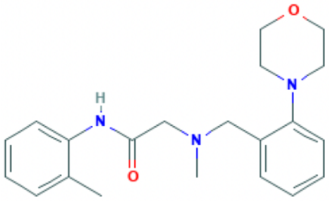
**Z126459618**	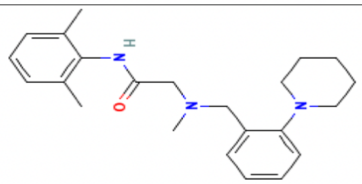
**Z126459802**	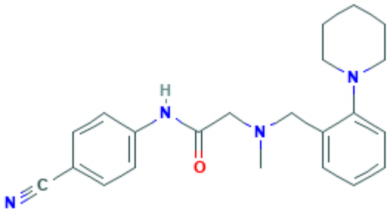
**Z126459820**	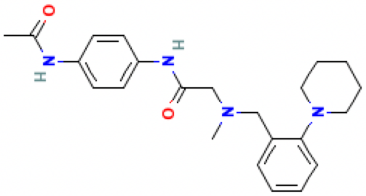
**Z126459924**	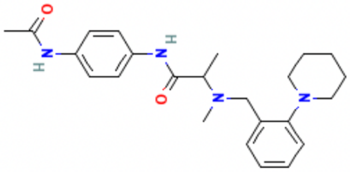
**Z136919828**	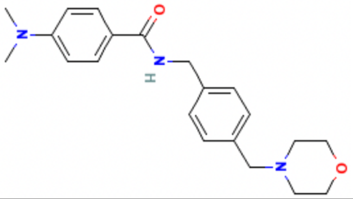
**Z169664026**	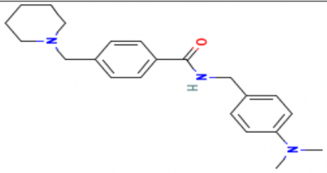

**Z188933242**	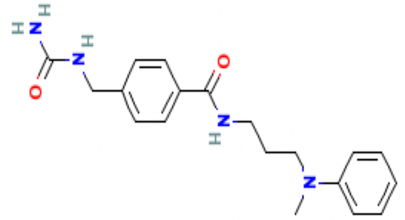
**Z191176110**	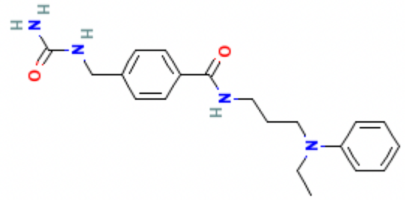
**Z195771716**	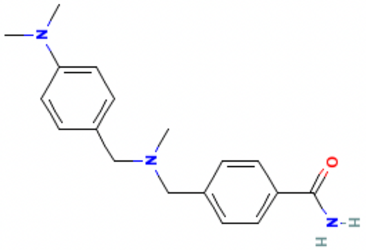
**Z27259780**	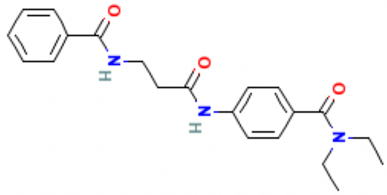
**Z27613695**	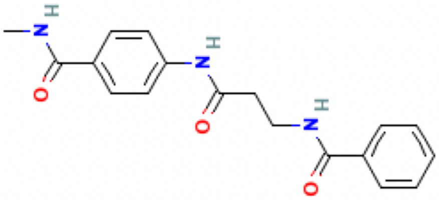
**Z27652642**	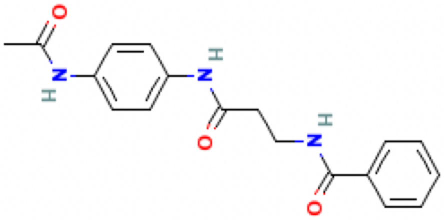
**Z48988699**	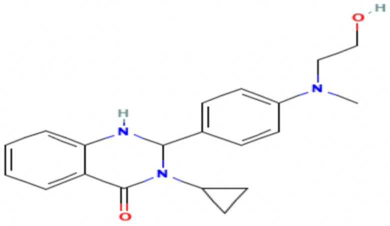

**Z62844532**	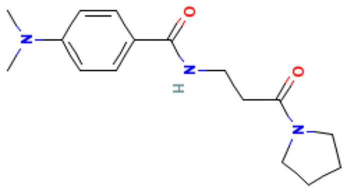
**Z74764931**	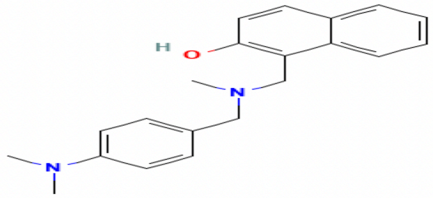
**Z88589564**	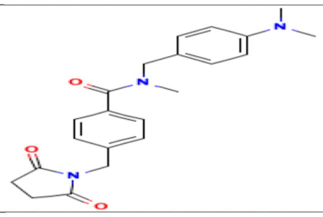
**Z89218846**	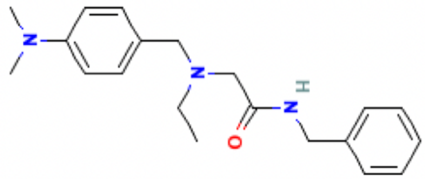
**Z91130744**	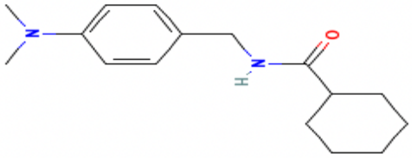
**Z91132478**	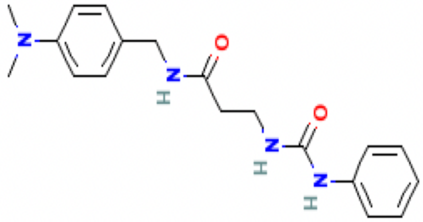
**Z91133559**	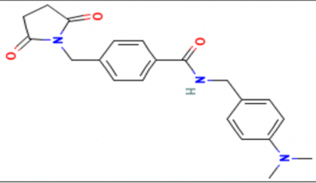
**Z95955130**	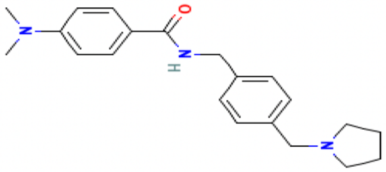

**Z27610715**	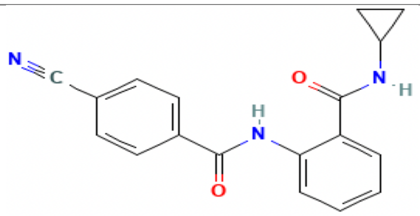
**Z27610667**	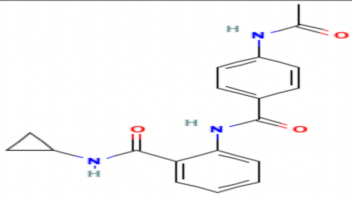
**Z109854354**	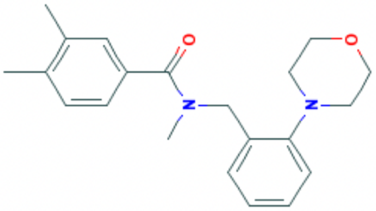
**Z109854260**	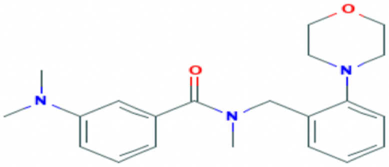
**Z126460086**	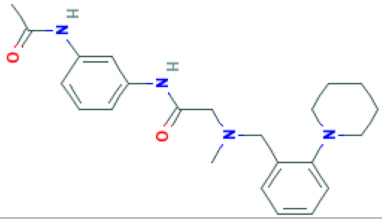
**Z126459548**	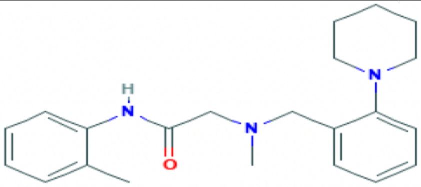
**Z126459614**	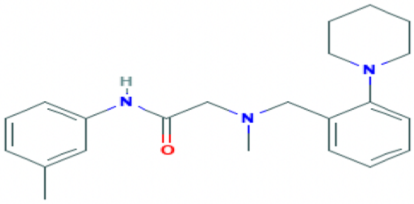
**Z126459742**	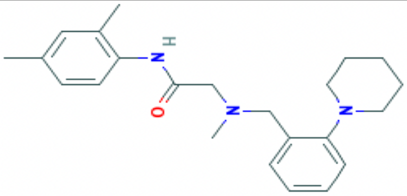

**Z432066854**	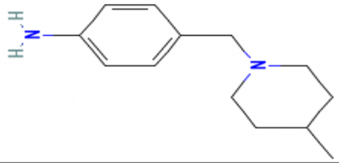
**BAS 01376666**	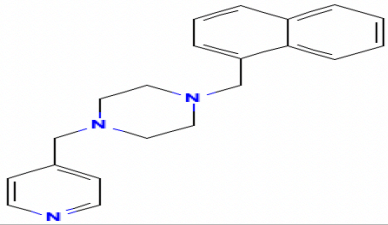
**ASN 04887130**	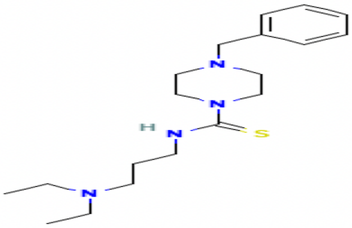
**BAS 06103177**	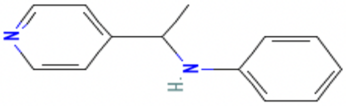
**BAS 06103407**	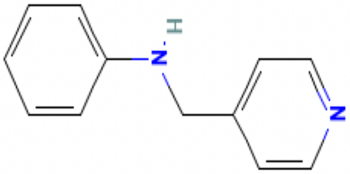
**BAS 07296990**	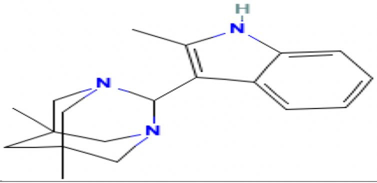
**BAS 01313325**	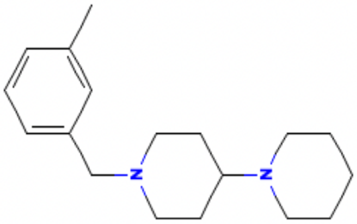
**BAS 06348345**	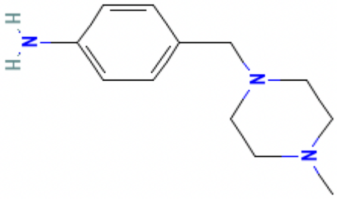

**BAS 06844821**	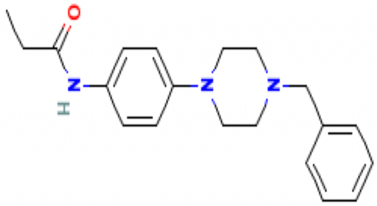
**BAS 07018849**	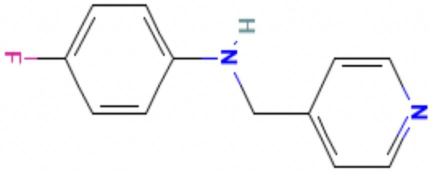
**BAS 09681156**	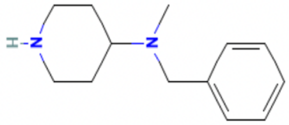
**BAS 07019774**	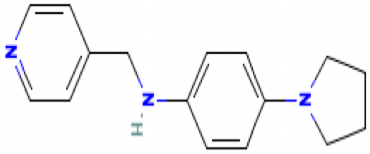
**BAS 06348344**	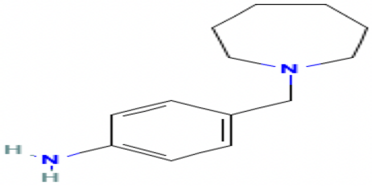

Supplemental Table S2. Top 12 predicted poses for unbiased docking of BAS 07019774 to GLI1 ZF.

Pose	Number of interactions	Residues[a]	Types of interactions	Energy	Distance A	Score
1	0	NA	NA	NA		-4.5157
2	2	Glu 334<br>His335	H-bond<br>Pi-Pi interaction	- 1.6 Kcal	2.99<br>3.61	-4.4947
3	1	Glu334	H-bond	-0.8 Kcal	3.42	-4.2653
4	1	His335	Pi-Pi interaction	-0.0 Kcal	3.96	-4.2068
5	1	Glu334	H-bond	-1.7 Kcal	3.11	-4.1595
6	1	DT A8	H-donor	-1.3Kcal	3.30	-4.0843
**7**	**3**	**Arg348**<br>**Glu334**<br>**His335**	**H-bond**<br>**Pi-H interaction**<br>**Pi-H interaction**	**-1.8 Kcal**	**3.31**<br>**3.72**<br>**3.30**	**-4.0129**
8	2	Arg 348<br>His 335	H-bond<br>H-pi interaction	-2.0 Kcal	3.06<br>3.91	-3.9755
9	0	NA	NA	NA		-3.9617
10	1	DC A7	Pi-H interaction	-0.9 Kcal	3.53	-3.9410
11	2	Arg 348<br>DT A8	H-bond<br>H-bond	-2.5 Kcal	3.16<br>3.5	-3.9080
12	1	His335	Pi-H interaction	-1.2 Kcal	3.58	-3.8048

[a] Residue numbering is for full length GLI1 (UNIPROY P08151). Residue numbering in poses below is as found in the Protein Data Bank for 2GLI. To convert to numbering for full length GLI1 (UNIPROY P08151) add 131.

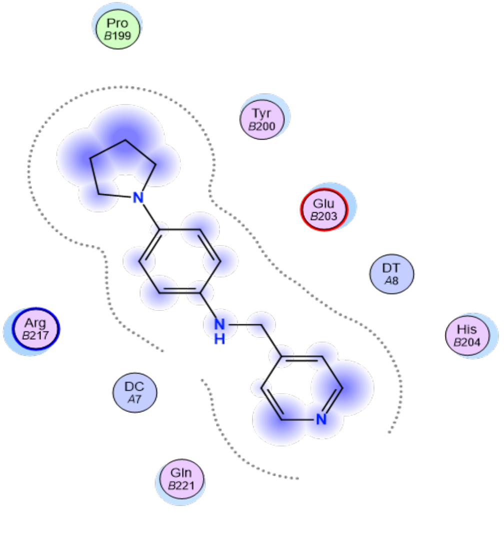

Pose 1

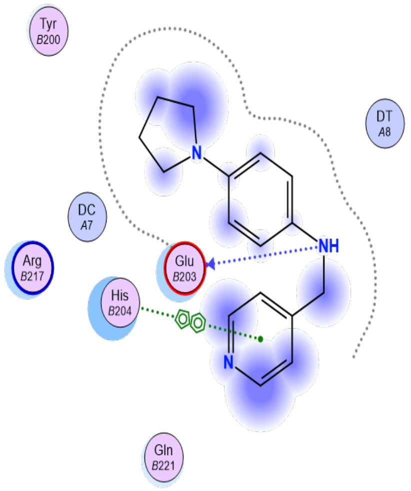

Pose 2

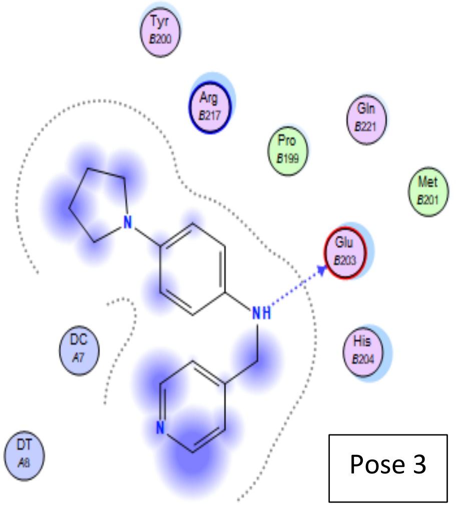

Pose 3

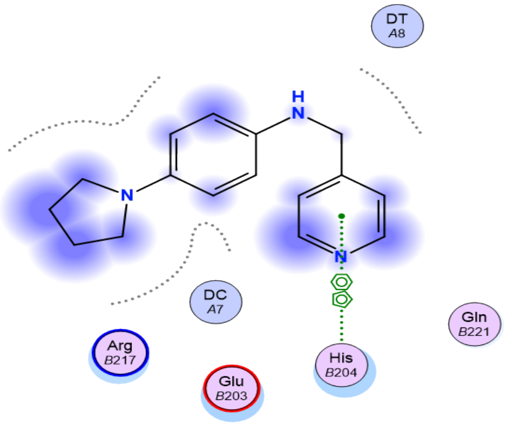

Pose 4

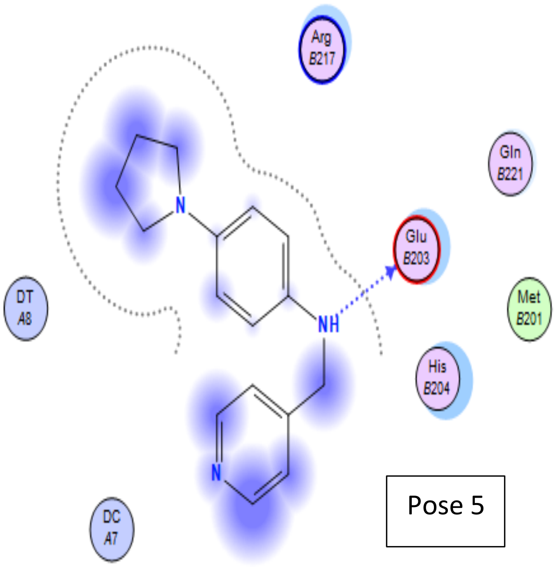

Pose 5

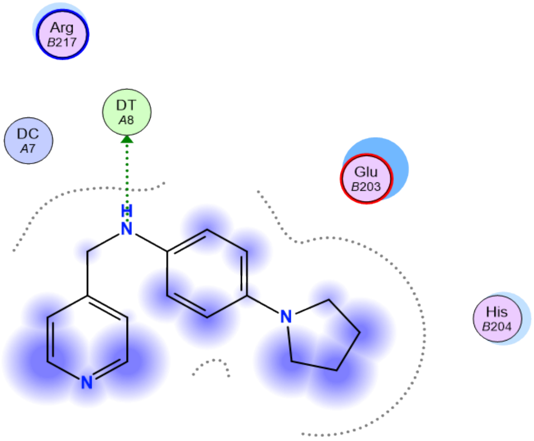

Pose 6

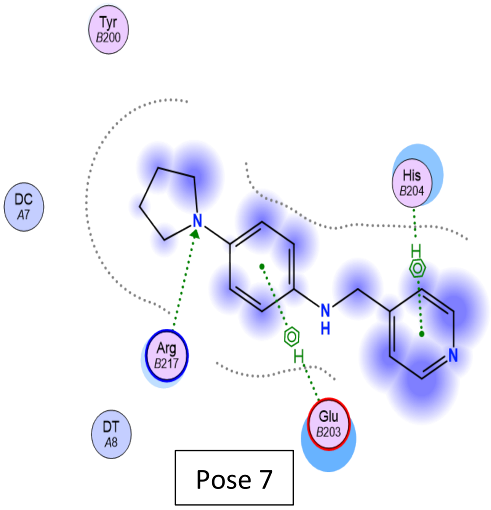

Pose 7

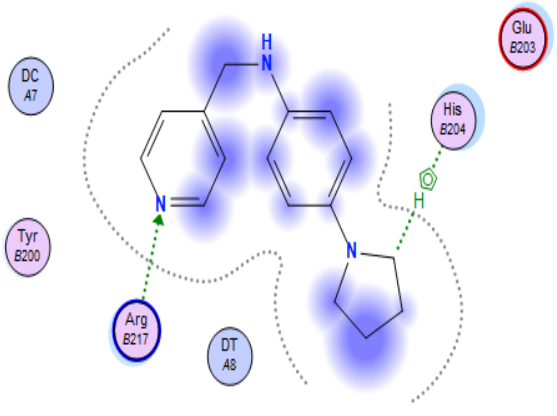

Pose 8

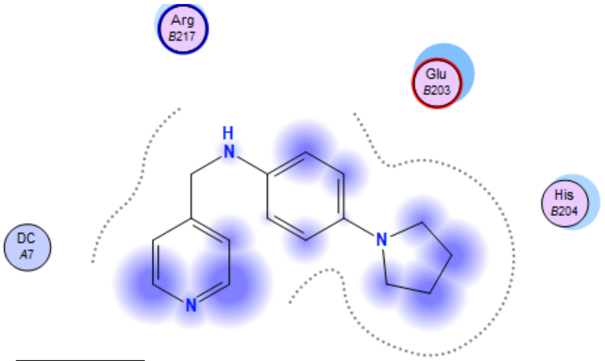

Pose 9

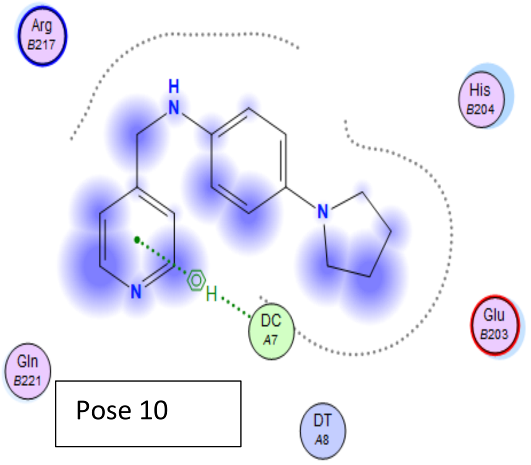

Pose 10

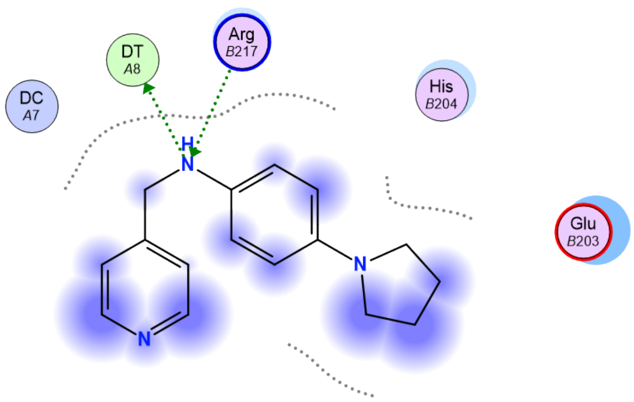

Pose 11

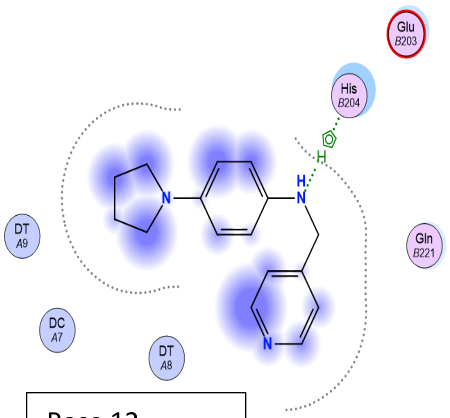

Pose 12

Supplemental Table S3. Top 15 predicted poses for biased docking of BAS 07019774 to GLI1 ZF at Glu119/Glu167.

(A)

File Edit Display Compute Window Help SVL DBV MOE Cancel

	mol	rseq	mseq	S	rmsd	rmsd_refine	E_conf	E_place	E_sc
1	1087685	1	1	-5.2245	31.8559	2.0207	-21.2643	-52.4070	-7
2	1087685	1	1	-5.2229	31.8953	1.4026	-21.3303	-56.0397	-7
3	1087685	1	1	-5.1398	30.0399	1.2291	-20.9146	-47.0204	-7
4	1087685	1	1	-5.1094	31.9854	1.1513	-19.1667	-56.5847	-8
5	1087685	1	1	-5.1079	32.9078	1.6559	-19.7336	-53.7915	-7
6	1087685	1	1	-5.0130	30.7462	1.1695	-19.6123	-51.3568	-7
7	1087685	1	1	-4.9935	34.0132	1.8567	-15.8517	-55.5493	-8
8	1087685	1	1	-4.9813	32.6022	1.3722	-20.3066	-56.7351	-7
9	1087685	1	1	-4.9761	31.4409	2.3126	-20.1758	-50.8227	-7
10	1087685	1	1	-4.9564	31.6166	2.0501	-20.5946	-46.8090	-7
11	1087685	1	1	-4.9324	31.2390	2.0174	-21.4409	-46.9732	-7
12	1087685	1	1	-4.8921	30.8040	2.2614	-17.3607	-50.7861	-7
13	1087685	1	1	-4.8878	31.9322	0.7939	-19.8852	-60.2349	-7
14	1087685	1	1	-4.8399	33.6236	2.3172	-21.4713	-59.1381	-7
15	1087685	1	1	-4.7815	31.6037	1.6934	-20.0873	-58.1580	-7

(B)

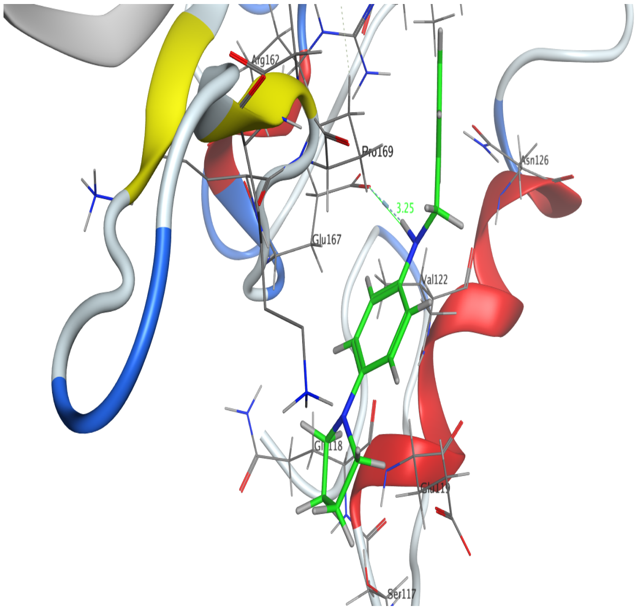

(C)

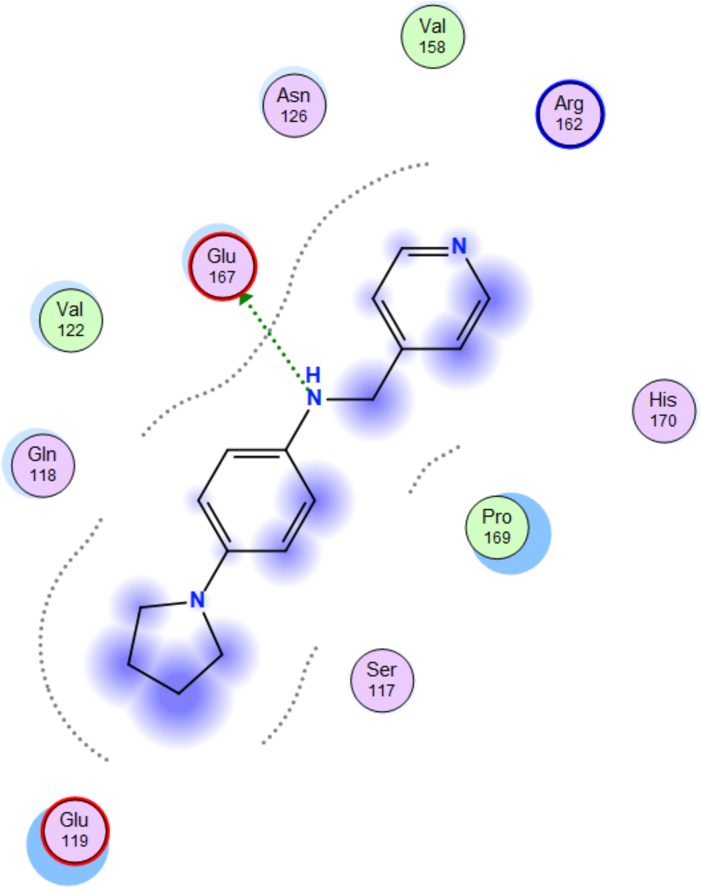

For docking in MOE, a grid was set at Glu119/Glu167. (B) 3D and (C) 2D best pose for of BAS 07019774 interacting at Glu119/Glu167 site.

Residue numbering is as found in the Protein Data Bank for 2GLI. To convert to numbering for full length GLI1 (UNIPROY P08151) add 131.
